# OpticalBERT and OpticalTable-SQA: Text- and Table-Based Language Models for the Optical-Materials Domain

**DOI:** 10.1021/acs.jcim.2c01259

**Published:** 2023-03-20

**Authors:** Jiuyang Zhao, Shu Huang, Jacqueline M. Cole

**Affiliations:** † Cavendish Laboratory, University of Cambridge, J. J. Thomson Avenue, Cambridge CB3 0HE, U.K.; ‡ ISIS Neutron and Muon Source, Rutherford Appleton Laboratory, Harwell Science and Innovation Campus, Didcot, Oxfordshire OX11 0QX, U.K.

## Abstract

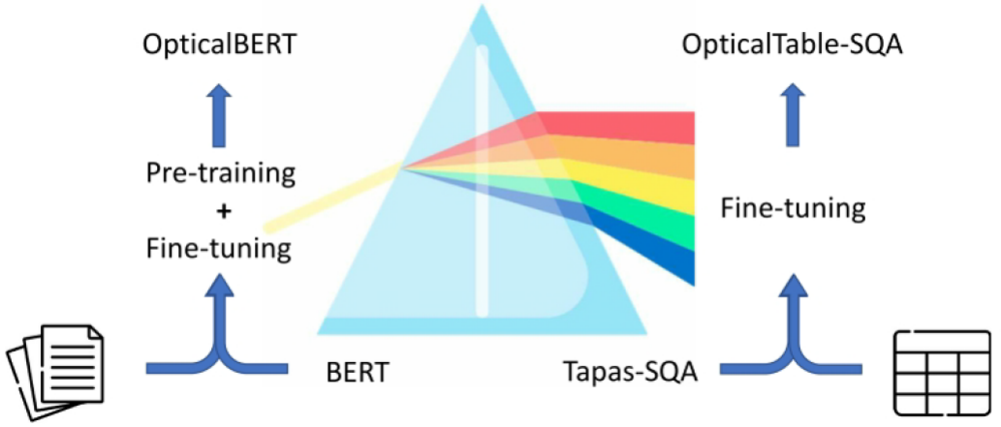

Text mining in the optical-materials domain is becoming increasingly important as the number of scientific publications in this area grows rapidly. Language models such as Bidirectional Encoder Representations from Transformers (BERT) have opened up a new era and brought a significant boost to state-of-the-art natural-language-processing (NLP) tasks. In this paper, we present two “materials-aware” text-based language models for optical research, OpticalBERT and OpticalPureBERT, which are trained on a large corpus of scientific literature in the optical-materials domain. These two models outperform BERT and previous state-of-the-art models in a variety of text-mining tasks about optical materials. We also release the first “materials-aware” table-based language model, OpticalTable-SQA. This is a querying facility that solicits answers to questions about optical materials using tabular information that pertains to this scientific domain. The OpticalTable-SQA model was realized by fine-tuning the Tapas-SQA model using a manually annotated OpticalTableQA data set which was curated specifically for this work. While preserving its sequential question-answering performance on general tables, the OpticalTable-SQA model significantly outperforms Tapas-SQA on optical-materials-related tables. All models and data sets are available to the optical-materials-science community.

## Introduction

Modern optical devices rely on optical materials. Based on how they interact with electromagnetic waves, different materials have been used to make various optical applications. For example, windscreens and optical lenses are often made of glassy materials since this affords their good light transmission; materials with certain light-absorption characteristics can be used to make optical filters.

Significant effort is being made in this field to accelerate novel materials development, in light of the growing demand for advanced optical devices of this nature.^1−3^ In recent years, natural-language processing (NLP) and its downstream tasks have been employed as powerful tools to extract materials-science information from textbooks, scientific publications, reports, and handbooks, to speed up the discovery of new materials. For instance, the “chemical-aware” text-mining tool ChemDataExtractor^4,5^ has been developed to autogenerate custom databases for materials-science applications. ChemDataExtractor has already been deployed to autogenerate bespoke databases for battery materials,^6^ Curie and Néel temperatures of magnetic materials,^7^ refractive indices and dielectric constants of optical materials,^8^ UV/vis absorption spectral attributes of materials,^9^ photovoltaic materials and their cognate device-performance characteristics,^10^ the band gap of semiconductors,^11^ and materials-engineering properties.^12^ These rule-based and machine-learning approaches have been complemented by several studies which have shown that unsupervised pretraining language models on large corpora can significantly improve performance on many generic, i.e., nonscientific, NLP tasks. These language models have been created using Long–Short-Term-Memory-based (LSTM) architectures, such as Embeddings from Language Models (ELMo),^13^ or transformer-based architectures, such as Generative Pretrained Transformer (GPT)^14^ and Bidirectional Encoder Representations from Transformers (BERT).^15^ However, directly applying these NLP methodologies to the optical-materials-science domain is not viable, since the vocabulary of general corpora (e.g., Wikipedia) and scientific publications about optical materials is quite different. Researchers have developed specialist BERT-based language models for the general scientific fields of biology (BioBERT^16^) and materials sciences (MatSciBERT^17^ and MatBERT^18^). Furthermore, a BERT-based language model that has been designed for a specific area of biology or materials science will naturally be more powerful than a general scientific BERT-based language model, as has recently been demonstrated through the presentation of BatteryBERT.^19^ We further advocate that property-specific BERT-based language models in materials science are needed as information sources for data-driven materials discovery, rather than general BERT-based models. Given that data-driven materials discovery is one of our key goals and very much on the agenda of the Materials Genome Initiative,^20^ we herein present a property-specific BERT-based language model for optical materials.

Furthermore, all aforementioned BERT-based language models have been exclusively trained on text, while previous work has emphasized the richness of information on optical properties in tables.^8^ NLP efforts that aim to extract information from tabular data include rule-based approaches such as TableDataExtractor^5^ and neural-network-based approaches such as TableQA (Table Question Answering),^21^ Tapas (Weakly Supervised Table Parsing via Pretraining),^22^ and T3QA (Topic Transferable Table Question Answering).^23^ The Tapas model developed by Google Research is worthy of particular mention since it significantly improved the state-of-the-art performance of three open table-based question-answering data sets.^22^ Nevertheless, these models were trained from general corpora (e.g., WikiTable), and applying such pretrained models to the optical-materials-science domain does not afford tractable results. First, a large number of tables in publications about optical-materials science include symbols that represent certain optical properties in the table header. For example, “nD” refers to the refractive index, and “ϵ” denotes the dielectric constant. None of these aforementioned neural network-based models can correctly understand these symbols. Additionally, most of the contents of table cells within general corpora (e.g., WikiTable) are text,^22^ while most of the contents of table cells within optical-materials-science publications are numbers, which can be a problem for developing a question-answering system that engages with tabular information from papers about optical materials.

Accordingly, this study seeks to realize new BERT-based models and table-based data-extraction capabilities that serve optical-materials research communities. Specifically, we develop OpticalBERT and OpticalPureBERT language models that are based on the BERT architecture but are trained on a large corpus of publications about optical materials. We examine the performance of these new models extensively by comparing their test results with those of the models that are designed for general use on three downstream tasks: abstract classification, question-answering, and chemical-named-entity recognition. We also develop the OpticalTable-SQA language model which serves as a question-answering tool for tabular data sources. This OpticalTable-SQA model has been realized by fine-tuning the Tapas model^22^ using a manually annotated data set of more than 4,000 question-answering pairs that pertain to the optical-materials domain. The OpticalTable-SQA tool demonstrates a significant improvement in understanding symbols of common optical properties, without a loss in the question-answering precision of the Tapas model on general table data sets.

## Methods

### Background

The BERT^15^ model has a transformer-based^24^ architecture. Instead of the traditional left-to-right language-modeling procedure, BERT achieves bidirectional information propagation by predicting randomly masked tokens in sentences and predicting whether or not two sentences follow each other (Next Sentence Prediction, NSP). In this study, we only used the former part of the BERT model architecture, i.e., its masked language model, since most downstream tasks that can make use of language models in the materials-science domain do not rely on NSP.

The Tapas model^22^ was also employed in this work, which uses the same structure as BERT for masked language modeling. Its token embeddings are combined with four more table-aware structural embeddings before feeding them to the language model, i.e., segment embeddings, column embeddings, row embeddings, and rank embeddings. These additional embeddings help to encode the structure of the table and enable the ultimate language model to correctly select table cells that contain the information sought once the model has been fine-tuned by a question-answering task.

### Corpus

**Figure 1:**
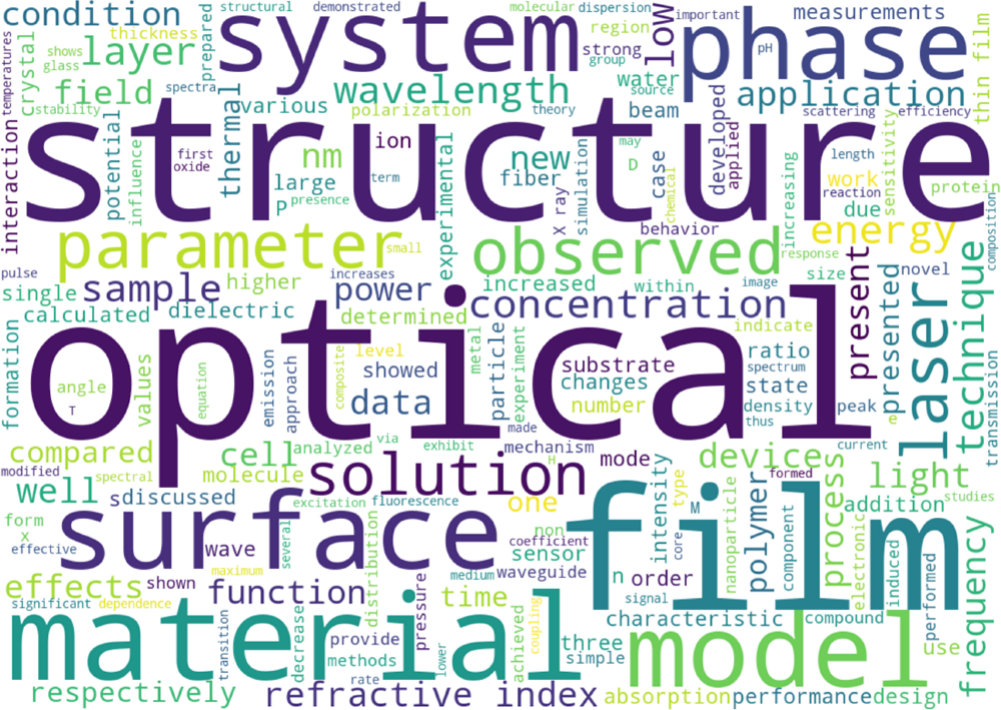
Word cloud of the most frequent words in the vocabulary of our corpus of papers about optical materials.

A total number of 668,188 papers were downloaded from the Royal Society of Chemistry (RSC) directly and from the Elsevier Science Direct using its sanctioned Application Programming Interface (API), with the queries keyword “optical material”. More details of the article-retrieval process can be found in a previous work reported by Zhao and Cole.^8^ The average paper contained 4,374 tokens, which is significantly larger than that of the 2,769 tokens which make up the papers that provided the data source for SciBERT.^25^ The overall corpus size used to create our language models for optical materials was 2.92B tokens, similar to the 3.3B tokens on which the originally crafted BERT model, BERT-base, was trained for generic-language (i.e., nonscientific) text, and similar to the 3.17B tokens on which SciBERT was trained. A word cloud that was generated from the abstracts of a random sample of 1,000 papers in our corpus is shown in Figure 1.

### Vocabulary

We constructed OpticalVocab, a new WordPiece vocabulary from our corpus about optical-materials science using the BertTokenizerFast library.^26^ The library uses the WordPiece tokenizer^27^ to collect the most frequently used words or subword units. Compared with a full-word dictionary or a character-level vocabulary, this subword tokenization method achieves an optimal balance between the size and the expression capability of the vocabulary.^27^ We used OpticalVocab in the creation of our OpticalPureBERT model, while the vocabulary file used to generate our OpticalBERT model was the same as that was used to create the BERT-base model.

**Figure 2:**
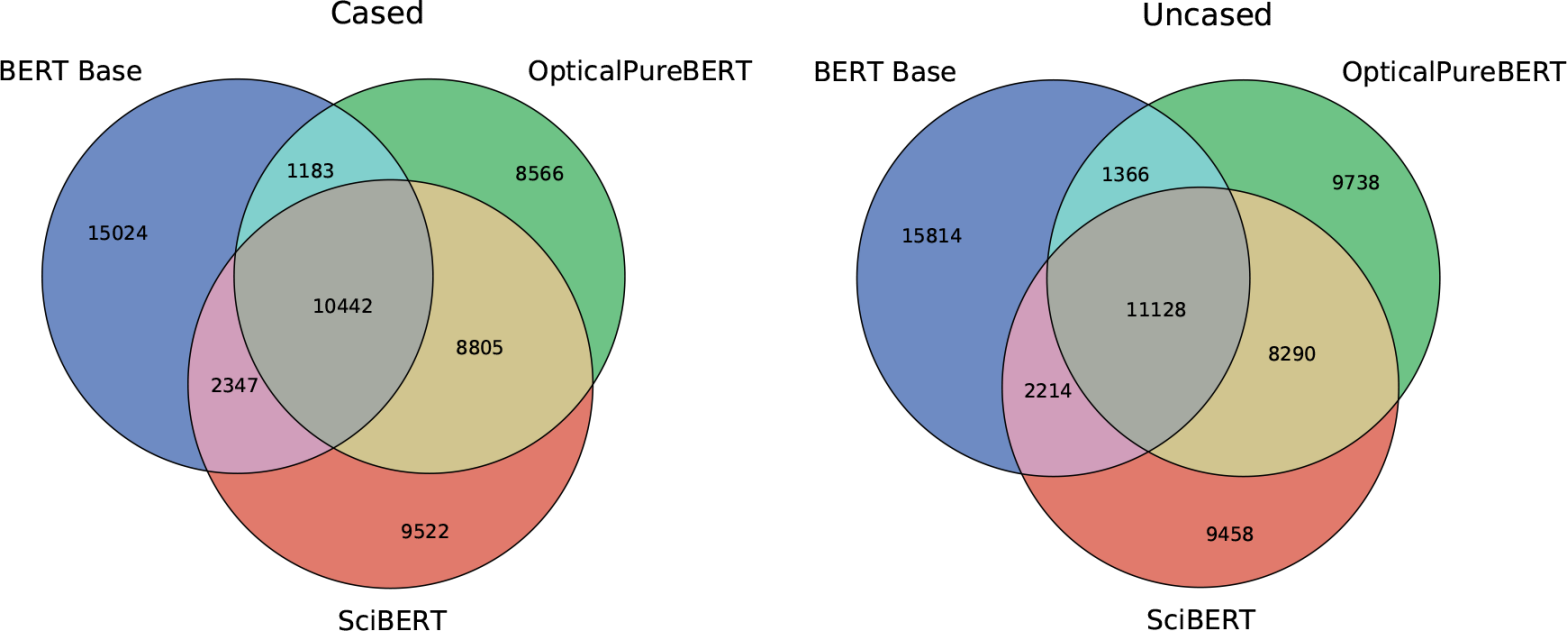
Comparison of vocabularies for the BERT-base, SciBERT, and OpticalPureBERT models. The digits represent the numbers of tokens in the corresponding vocabularies.

The quality of our vocabulary and tokenizer was evaluated by plotting Venn diagrams^28^ of vocabularies that pertain to the BERT-base model, the SciBERT model, and our OpticalPureBERT model, as shown in Figure 2.

**Figure 3:**
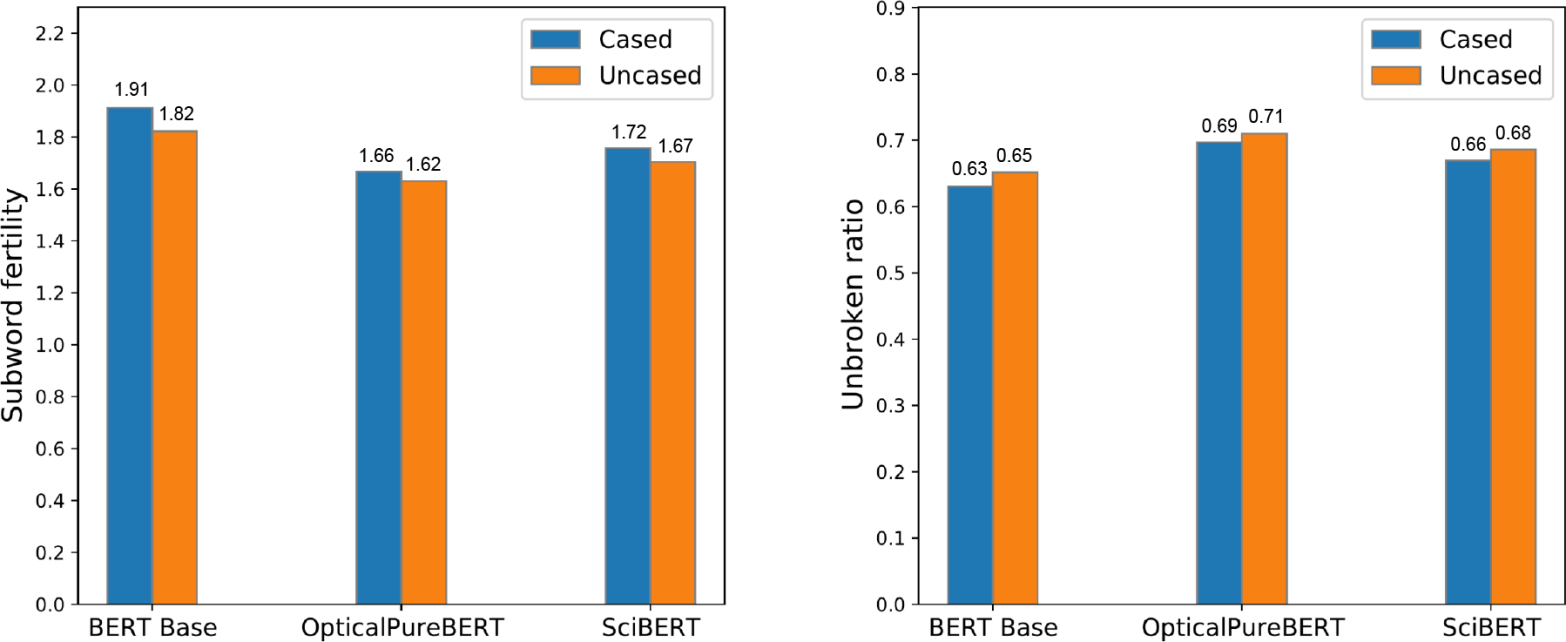
Subword fertility (lower is better) and unbroken ratio (higher is better).

The largest token overlap between OpticalVocab and the other two vocabularies is 66.4% (cf. the intersection between the cased OpticalPureBERT and SciBERT models), which illustrates a substantial difference in frequently used words between text in papers about optical-materials science and about general science topics. We also show the subword fertility^29^ and the unbroken ratio^30^ of these three tokenizers in Figure 3. The subword fertility measures the average number of subwords that have been created after a word has been tokenized. The unbroken ratio counts the fraction of words whose completeness is preserved after tokenization.

Figure 3 shows that the OpticalVocab used by OpticalPureBERT reduces the splitting of words when compared with the vocabularies of the other two models. This suggests that OpticalVocab is better suited for downstream tasks on our optical-materials-science corpus.

### Pretraining

**Figure 4:**
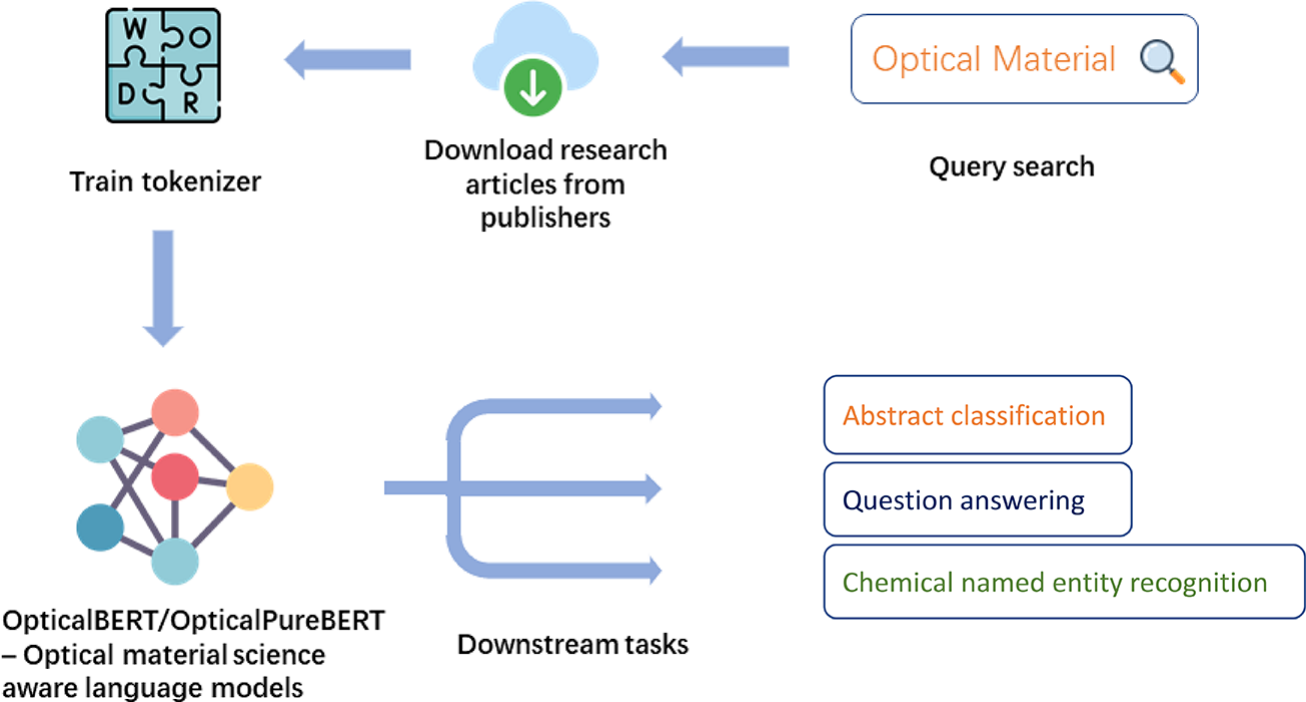
Four overarching stages of the optical-materials-related BERT model development: corpus construction, tokenizer training, pretraining, and fine-tuning.

Figure 4 shows the four overarching stages in which the two optical-materials-related BERT models were developed. In the pretraining stage, the OpticalBERT model was trained on our optical-materials corpus after initializing weights from the BERT-base model, while the OpticalPureBERT model was trained from scratch on the same corpus using the same architecture as the BERT-base model. We trained two different versions of the OpticalBERT and OpticalPureBERT models: cased and uncased. The cased models were pretrained using the raw corpus, while uncased models were pretrained using the corpus where the characters of all words were confined to their lowercase. Masked-language modeling (MLM) was used as the primary training phase of the two models, in which 15% of words in the employed corpus were masked and the model was trained to predict the masked words. We trained all of our models with a batch size of 256 sequences and a maximum sequence length of 512 tokens. The required training time was 8 days for OpticalBERT models (further trained for 35 epochs from BERT weights) and 10 days for the OpticalPureBERT models (trained for 40 epochs), using eight NVIDIA DGX A100 GPUs on the ThetaGPU cluster at the Argonne Leadership Computing Facility (ALCF). Details of the pretraining hyperparameters can be found in the Supporting Information. All of our models were implemented in PyTorch^31^ using transformers.^26^

### Fine-Tuning

Our optical-materials-related BERT models can be applied to various downstream tasks with minimal changes to the BERT architecture. Thereby, we fine-tuned our OpticalBERT and OpticalPureBERT models on the following three text-mining tasks that are relevant to materials science: Abstract Classification, Question Answering (QA), and Chemical-Named-Entity Recognition (CNER).

#### Abstract Classification

Abstract/document classification refers to the task of classifying whether or not the abstract of a given research paper is relevant to a given field. In this study, we fine-tuned the pretrained BERT-based models by adding a new sequence-classification layer on the top of them. The output of this layer will either be 1, i.e., this abstract or paper is relevant to the research field of optical-materials science, or 0, i.e., this abstract or paper is focusing on other subjects. As the search through papers in the scraping process was undertaken by simply finding the phrase “optical material” within a given paper, this corpus of papers will inherently include publications that are unrelated to optical-materials research. For example, the optical property of a lens could be mentioned in a paper that focuses on biomedical surgical operations, where the essence of that research is not about optical materials. Successful classification of these papers into those relevant to the field of optical-materials research, or otherwise, will not only help to improve the accuracy of data-extraction tasks that employ this corpus by filtering through only papers of true interest but also will save a lot of time in a high-throughput text-mining study.

Annotated data are difficult and costly to collect for a specific scientific field owing to the domain expertise that is required for high-quality annotation.^25^ However, we were able to build a training data set for the abstract classification task by selecting papers based on their journal names. Half of the data set contained papers that have “optic” in their journal names, such as “Optical Fiber Technology” and “Optical Materials”. The other half of the data set contained papers that were definitely not talking about optical materials. We selected this latter category of paper by excluding papers where it was difficult to determine whether they are talking about optical materials based on purely their journal names. A few examples of these “obscure” journal names are “Journal of Nanoparticle Research”, “Results in Chemistry”, and “Tetrahedron”. The resulting data set contains 17,748 abstracts that are believed to be relevant to optical-materials research and 17,748 abstracts that are believed to be irrelevant to optical-materials research, based solely on the journal name. The quality of this data set was validated by randomly sampling 300 abstracts and manually labeling whether or not they are correctly classified. A consistency ratio of 92.5% between manual labeling and “journal name labeling” suggests that our data set is of a sufficiently high quality to be used in an abstract-classification task. This small validation data set can be found in the Supporting Information.

The data set was divided according to a 80:20 split of a training set and a development set. We manually annotated another randomly sampled out-of-sample test data set of 315 arbitrary abstracts, i.e., abstracts whose journal name could belong to the ‘obscure’ category, in order to evaluate the real-world performance of our models. We fine-tuned the BERT-base model and the SciBERT model so that we could make a fair comparison of their performance against that of our new models. We also fine-tuned the MatBERT model^18^ and the MatSciBERT model^17^ on the same training set and used their performance on the same test sets as baselines, as they are existing BERT-based models that were also pretrained on materials science corpora. We likewise trained a logistic regression (LR)-based binary classification model as another baseline, so that we could compare the performance of our models against that of other techniques. Additionally, we tested the performance of zero-shot prompt learning^32^ on the test set.

#### Question-Answering Tasks on Text

In an (extractive) Question Answering (QA) process, questions are posed in natural language to a paragraph of a given research paper, and the algorithmic routines of the QA process aim to extract the correct answer to that question from the paragraph. QA tasks can be applied to BERT-based models by adding a linear layer to the BERT architecture at the top of its output, i.e., the sequence embedding. This linear layer generates two one-dimensional logits whose length corresponds to that of the sequence length: 1) the “start logits”, which are used to compute the probability of a given token to be the start of the predicted answer, and 2) the “end logits”, which are used to compute the probability of one token to be the end of the predicted answer. The confidence score, *S*, of a certain text slice, which starts at token *i* and ends at token *j*, to be the answer is given as
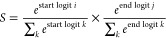
1

This can be also written as
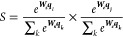
2where ***q***_***i***_, ***q***_***j***_, ***W***_***s***_, and ***W***_***e***_ are, respectively, the token embedding of the start token, the token embedding of the end token, and the weights of the linear layers for the start and end logits. In this way, the piece of text that starts at token *i* and ends at token *j* with the highest combined probability will be identified as the model output.

The SQuAD v1.1 data set^33^ was used to fine-tune our BERT-based models for QA applications. It is worth mentioning that the SQuAD v2.0 data set was not used in this fine-tuning process despite being released more recently than the SQuAD v1.1 database; this is because it contains unanswerable questions, which makes it less applicable to the science domain.^16,19,34^ The SQuAD v 1.1 data set contains about 100,000 question-answer pairs for machine comprehension in the general text domain. The data set was split in a 90:10 ratio for the purpose of its training and development, respectively.

**Figure 5:**
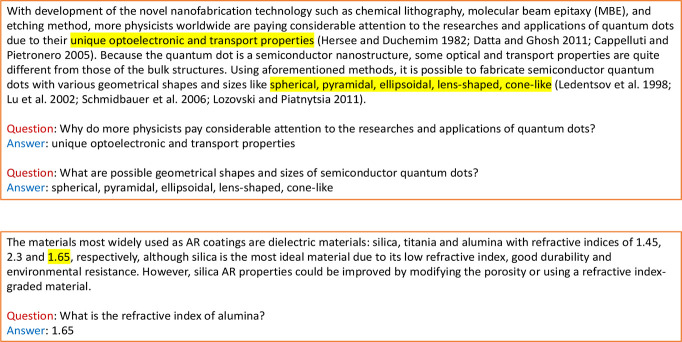
Example constructs of data entries for the evaluation question-answering test data sets. Top: arbitrary QA example. Bottom: numerical QA example. Annotated correct answers are highlighted.

To evaluate the real-world performance of the fine-tuned models, we created two manually annotated out-of-sample test sets. The first test set contains 301 arbitrary question-answer pairs from a randomly sampled set of 162 paragraphs which are not in our pretraining corpus. The second test set contains 317 numerical question-answer pairs from the corresponding 305 paragraphs that had been sampled from an existing database about optical materials that had been previously autogenerated by ChemDataExtractor.^8^ Example constructs of data entries for these evaluation test sets are shown in Figure 5. A comprehensive horizontal comparison is performed between the fine-tuned OpticalPureBERT model, the OpticalBERT model, the BERT-base model, and the SciBERT model, as well as the MatBERT model^18^ and the MatSciBERT model.^17^ We also compared the test predictions of the numerical data set with the original records in the ChemDataExtractor-generated database,^8^ from which this numerical data set was constructed.

#### Chemical-Named-Entity Recognition (CNER)

Chemical-named-entity recognition is one of the most fundamental steps that is required of the text-mining process when extracting data from the physics- or chemistry-related materials domain. This is because the primary identifier of the data sought is a chemical-named entity that needs to be recognized and hence extracted from a sentence of text. Conventional approaches to CNER have often focused on a hybrid combination of rule-based methods and LSTMs.^4,35,36^ Recent studies have significantly improved the performance of CNER on existing data sets by fine-tuning BERT-related models.^16,17,37^ By adding a single-output layer that is based on the word embedding of its last layer to the BERT architecture, BERT is able to compute its token-level probabilities within the BIO scheme.^38^ The BIO scheme assigns three types of labels to each token of a sentence, “B-MAT” which indicates that the token refers to the beginning of a chemical name, and “I-MAT” which suggests that the token is part of a chemical name but is not its starting token, while “O” classifies all other ordinary tokens. It is necessary to include both “B-MAT” and “I-MAT” labels, as words will be split into subword tokens by the BERT tokenizer.

The data sets that were used to fine-tune our models are a combination of the BioCreative IV CHEMDNER data set^39^ and the Matscholar data set.^40^ The CHEMDNER data set contains 84,355 manually annotated chemical-named entities that span across 10,000 abstracts, and it has an interannotator agreement of 91%. The majority of the chemical names in the CHEMDNER data set are organic, owing to the disciplines from which its source papers were selected.^37^ We thus included the Matscholar data set in our training data set together with the CHEMDNER data, in order to enhance the capability of our model, so that it can identify inorganic materials as well as organic ones. Although the 7,360 chemicals that have been annotated in the Matscholar data set are far fewer than those of the CHEMDNER data set (84,355), the Matscholar data set focuses on materials science which better suits our objectives. The combined data set was divided into training and development sets using the 80:20 split ratio, which is in common with data-splitting procedures that had been applied to assess other downstream tasks. A manually annotated out-of-sample test set was constructed to include 411 random-sampled examples of chemical-named entities. The performance of our models on the development set was compared with that of other BERT-based models, while the model performance on the test set was compared additionally with traditional NLP models that have been made via ChemDataExtractor.^5^

#### Question-Answering Tasks on Tables

We enabled question-answering capabilities for tabular data by focusing on teaching the model to understand various symbols of optical properties that reside in the header of tables which are contained within papers that make up the optical-materials-science corpus. This strategy was adopted because an optical property is commonly not presented precisely in complete English words within a table; rather, it is represented by a symbol of it. This task of selecting the correct cell from a table when the model is asked for a specific optical property of a certain compound, or a chemical in natural language, is known as a “single-cell extraction”. The publicly available table-parsing sequential question-answering model for general text, Tapas-SQA,^22^ provided a generic baseline model for our requirements which we could adapt for our scientific application. The Tapas-SQA model had been fined-tuned on the Sequential Question Answering (SQA)^41^ data set, which consists of 17,553 generic (i.e., nonscientific) question-answering pairs that had been annotated from tabular data on Wikipedia. The SQA data set is called a sequential question-answering data set because its annotation allows for a configuration where several questions might be asked of the data in series by a single enquirer. For example, a sequential set of questions asked by one enquirer could be

1. who are the players?
2. of those, who is from the USA?

The Tapas-SQA model is required to answer these questions in series, by taking the output of the first question as an input to answer the second question. The SQA data set^41^ has a preidentified test set, which enables direct comparisons between models that have been developed using different approaches. This test set contains 1,025 first questions, 1,024 s questions, 683 third questions, and 280 questions of higher orders. Thus, the ability of the Tapas-SQA model to correctly answer the first or first two questions plays a crucial role in real-world applications, where people often ask a series of interrelated questions in order to tackle a problem or to understand a situation.

**Figure 6:**
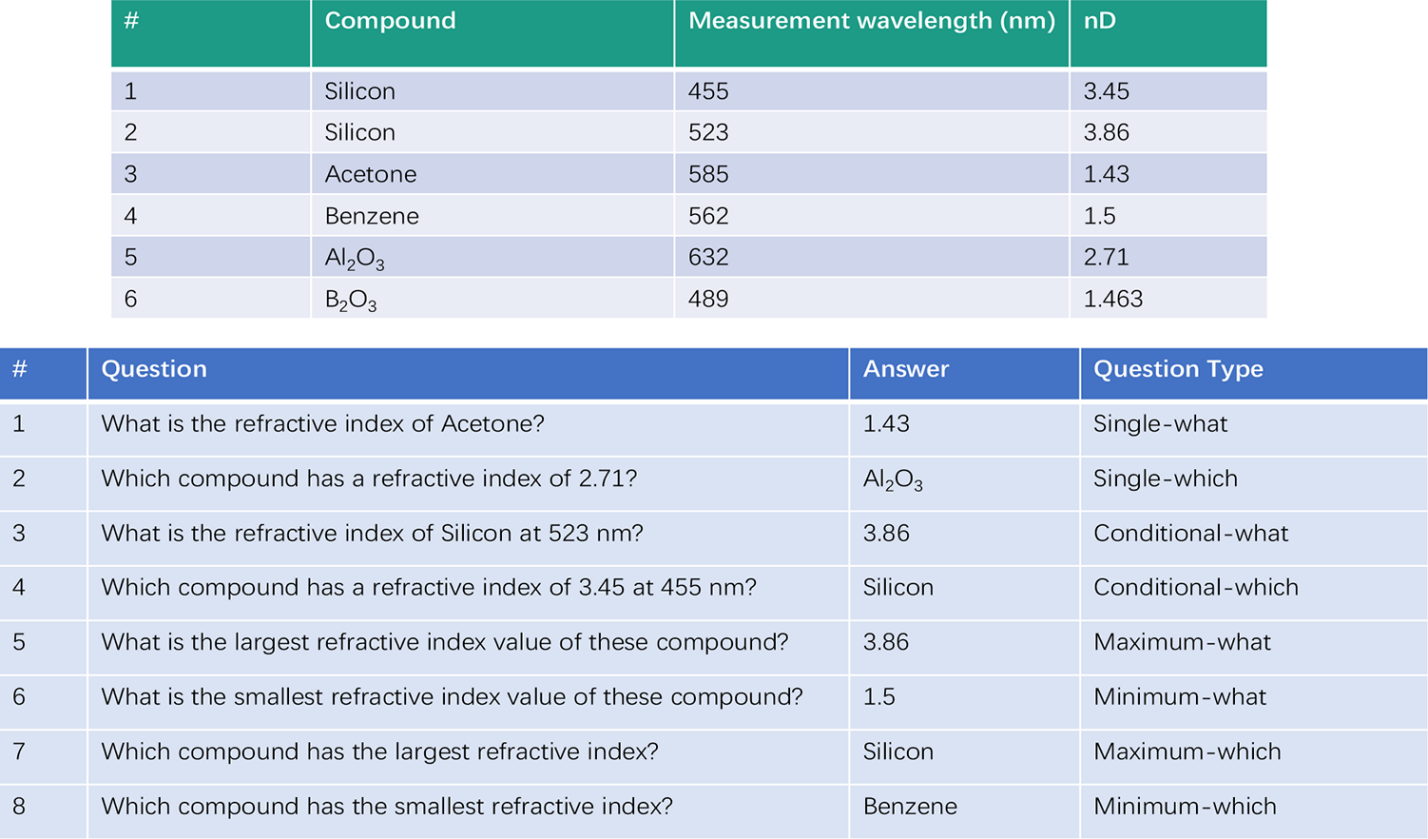
A table (top) with corresponding example question-answering annotations and corresponding question types (bottom).

**Figure 7:**
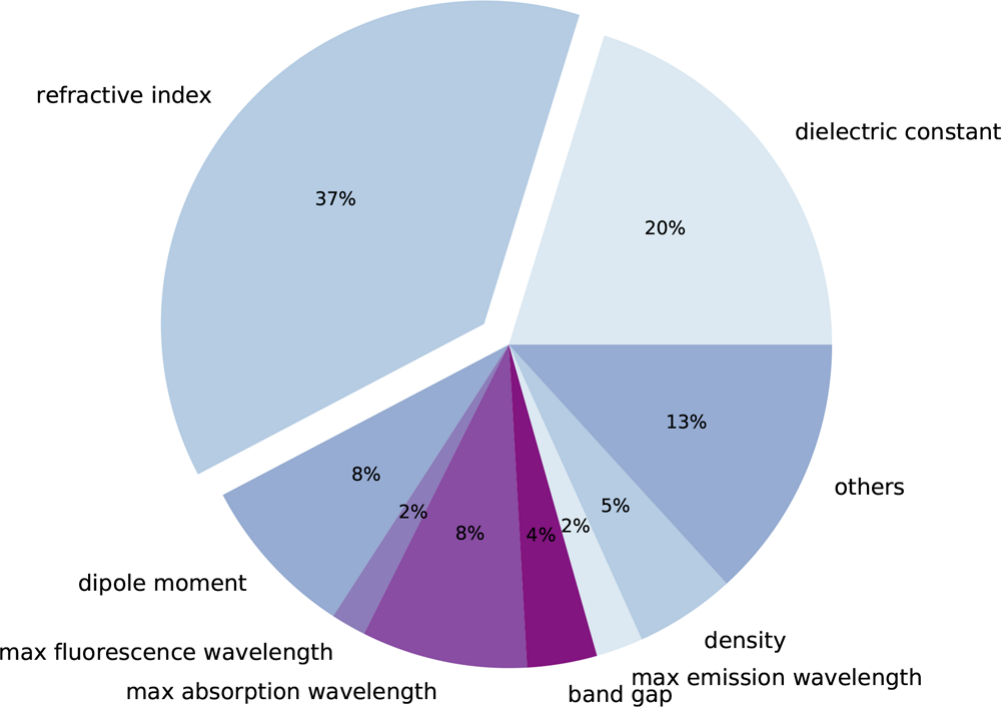
Percentage compositions of different (optical) properties that feature in our OpticalTableQA data set.

Both the original corpus that had been used to train the Tapas-SQA model and this SQA data set^41^ contain a very limited amount of information about the optical-materials-science domain. Thus, we sought to adapt the Tapas-SQA model to suit our application area, by augmenting it with optical information from tabular data. Thereby, we first created the OpticalTableQA data set, which contains 4,534 manually annotated single question-answering pairs on tabular data about optical materials from more than 90 tables, where “single” refers to the fact that all question-answering pairs are first questions. These tables were taken from the papers that make up the aforementioned corpus that was used to train the OpticalBERT model, whereby they were filtered based on their structure, length, and content, in order to meet the requirements for fine-tuning the Tapas-SQA model.^22^ We designed eight question types that can be classified into two categories: 1) “what-questions” which ask about the property value of a chemical or compound and 2) “which-questions” which require the Tapas-SQA model to select the correct chemical or compound given a property value. The eight question types and the annotation process are exemplified in detail in Figure 6. To enhance model generalizability, this question-answering annotation was designed to cover a wide range of optical properties including, but not limited to, the refractive index, dielectric constant, absorption/fluorescence maximum, band gap, and dipole moment. The percentage composition of physical properties that are featured in this OpticalTableQA data set is given in Figure 7.

We then fine-tuned the Tapas-SQA model on the OpticalTableQA data set to 1) build a model that is able to understand special symbols and certain table structures that pertain to the optical-materials-science domain and 2) preserve the ability to answer general questions of the adapted Tapas-SQA model as far as possible. The OpticalTableQA data set was shuffled and divided into training and development subsets whose proportioning carried an 80:20 ratio, respectively. This dividing procedure was implemented five times whereby the data set was split randomly in each case. Thus, five pairs of training and development subsets of the OpticalTableQA data set were created. For each of those five pairs of data subsets, the Tapas-SQA model was fine-tuned on the training set, and the performance of the fine-tuned model was evaluated using the development set. Meanwhile, the performance of the fine-tuned model on the SQA test set was compared with that of the original Tapas-SQA model.

## Results and Discussion

### Pretraining

We pretrained the OpticalPureBERT model for 40 epochs as it was being trained from scratch. This was more computationally intensive than the pretraining stage for the OpticalBERT model, which was only trained for 35 epochs, because it employed initialized weights from the original BERT-base model.^15^ Our OpticalPureBERT model employed our domain-specific vocabulary, OpticalVocab. Thus, its processed corpus was more compact than that of the OpticalBERT model which used the default BERT vocabulary. These two factors complemented each other in that the resulting number of training steps for our two models was comparable: ∼1,273,480 steps for OpticalPureBERT and ∼1,279,005 steps for OpticalBERT. For comparison, the BERT-base model was trained for 1 M steps.^15^

**Figure 8:**
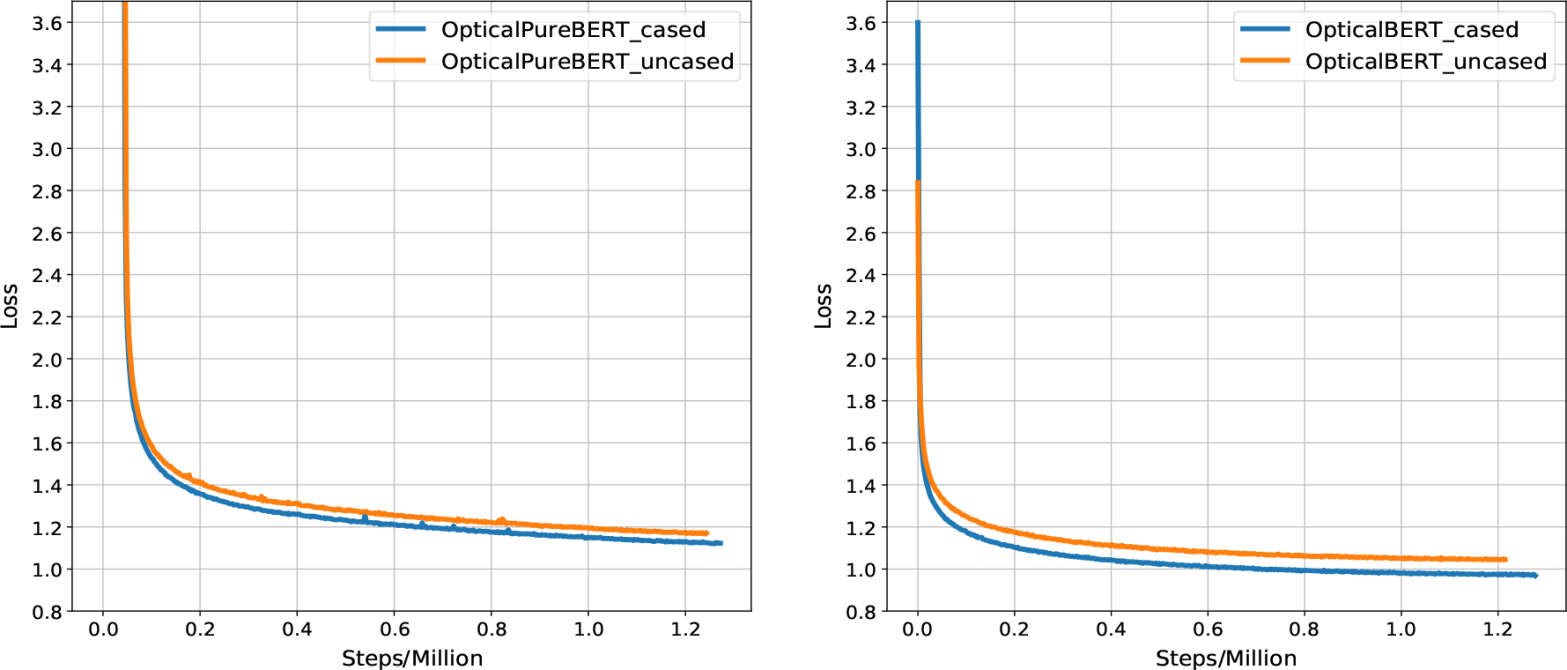
Training losses versus pretraining steps for OpticalPureBERT and OpticalBERT models.

Figure 8 shows the progressive change in training losses for our models with respect to the number of training steps that were employed during the pretraining processes. Overall, training losses for our cased models converged to a lower value than those of the uncased models. This is natural since capitalized characters offer additional information that is useful in predicting masked tokens. The training losses for the OpticalPureBERT models converged more slowly and to a larger value than those of the OpticalBERT models owing to the fact that the OpticalPureBERT models were trained from scratch. The efficiency of our final pretrained language models was evaluated according to their relative performance when applied to various optical-materials-science domain-specific downstream tasks, which will be covered in detail in the following sections.

### Fine-Tuning

**Figure 9:**
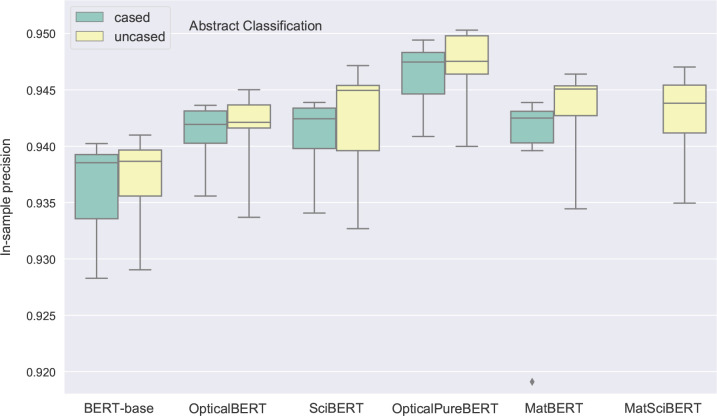
Box plots of the in-sample precision of 11 fine-tuned BERT-based models for cased and uncased models on the abstract classification development set. Each box represents the distribution of the model precision across six hyperparameter sets. The lower and upper quartiles of each distribution are denoted by the horizontal boundaries of a box, and the median value is signified by the horizontal line within a box. The minimum and maximum values of the range of the distribution are given by the top and bottom tails, while outliers that extend beyond the 4 * interquartile range are denoted by diamonds. Styles and annotations of the plot have the same meanings across Figures 9, 11, 13, and 15.

**Figure 10:**
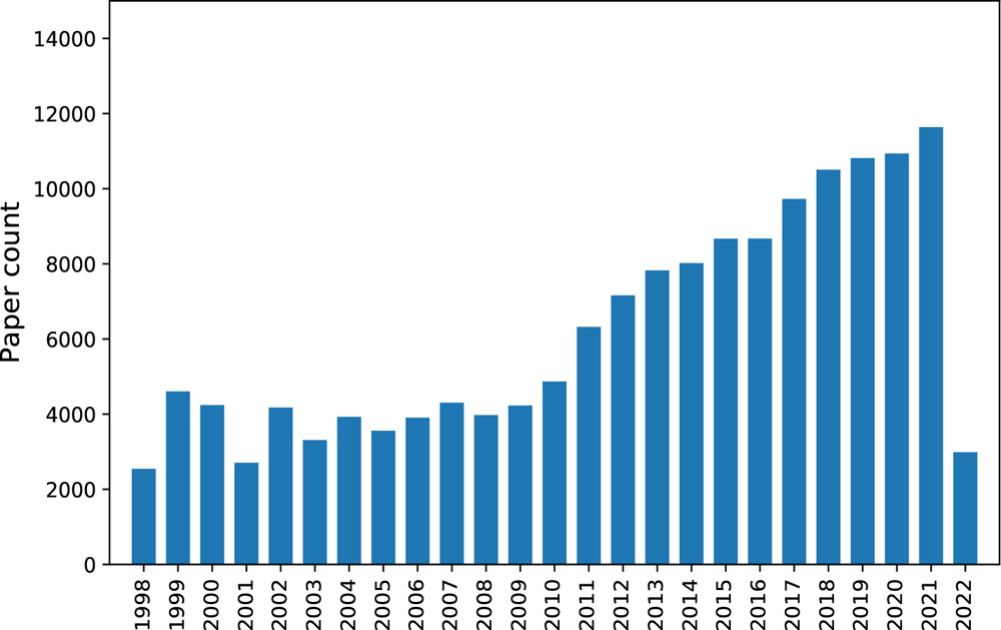
A bar chart showing the number of published papers about optical materials that were selected by our abstract classification model from publications that span the last 24 years. Paper records for 2022 included data only up to February 2022 (inclusive) as the data in this study were extracted up to this point.

**Figure 11:**
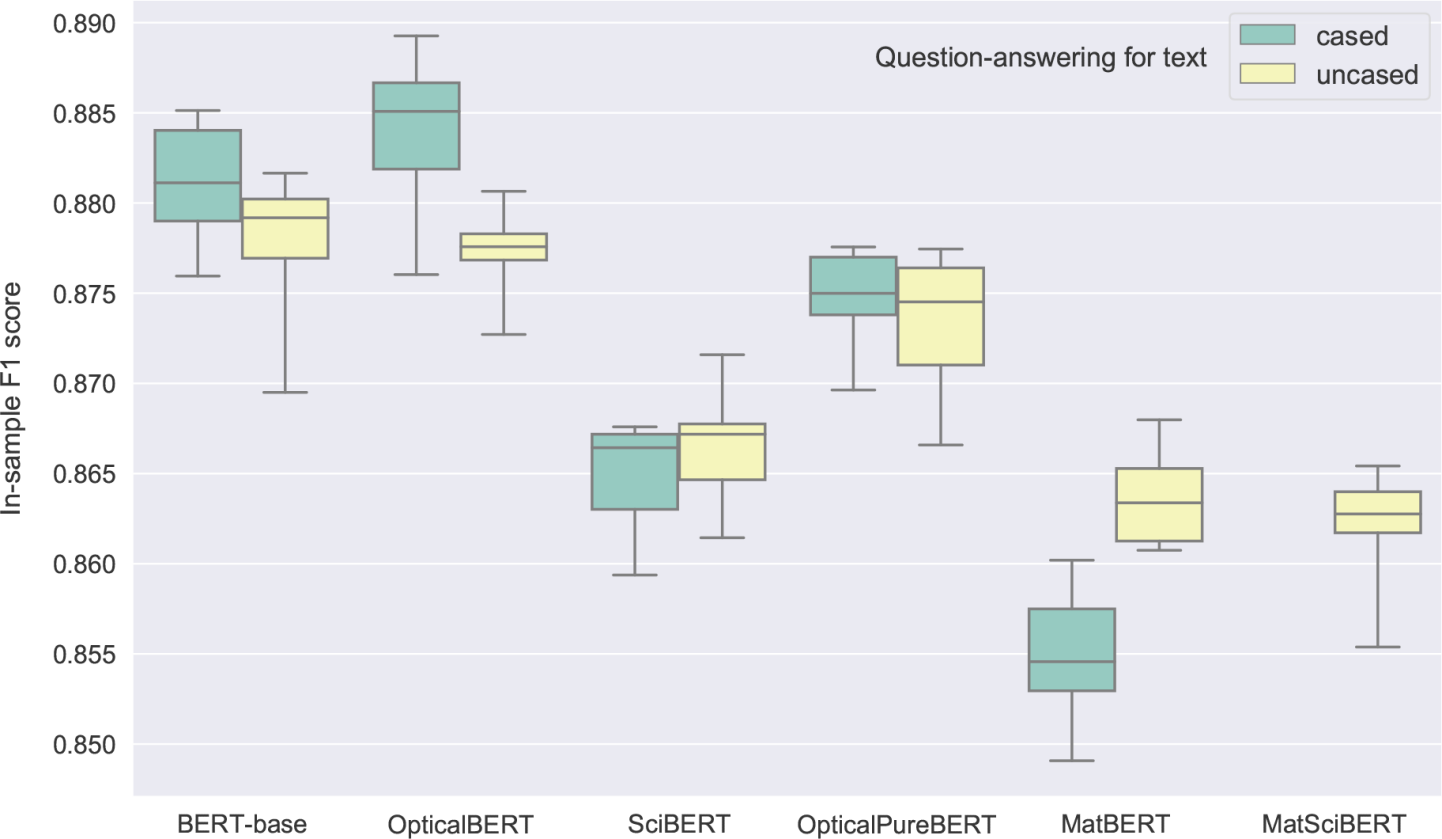
Box plots showing the distribution of F1 scores for 11 fine-tuned BERT-based models (5 cased and 6 uncased), when applied to the question-answering downstream task using the in-sample development set. Each box represents a distribution of six F1 scores of the model across different combinations of the learning rate, batch size, and epoch numbers.

**Figure 12:**
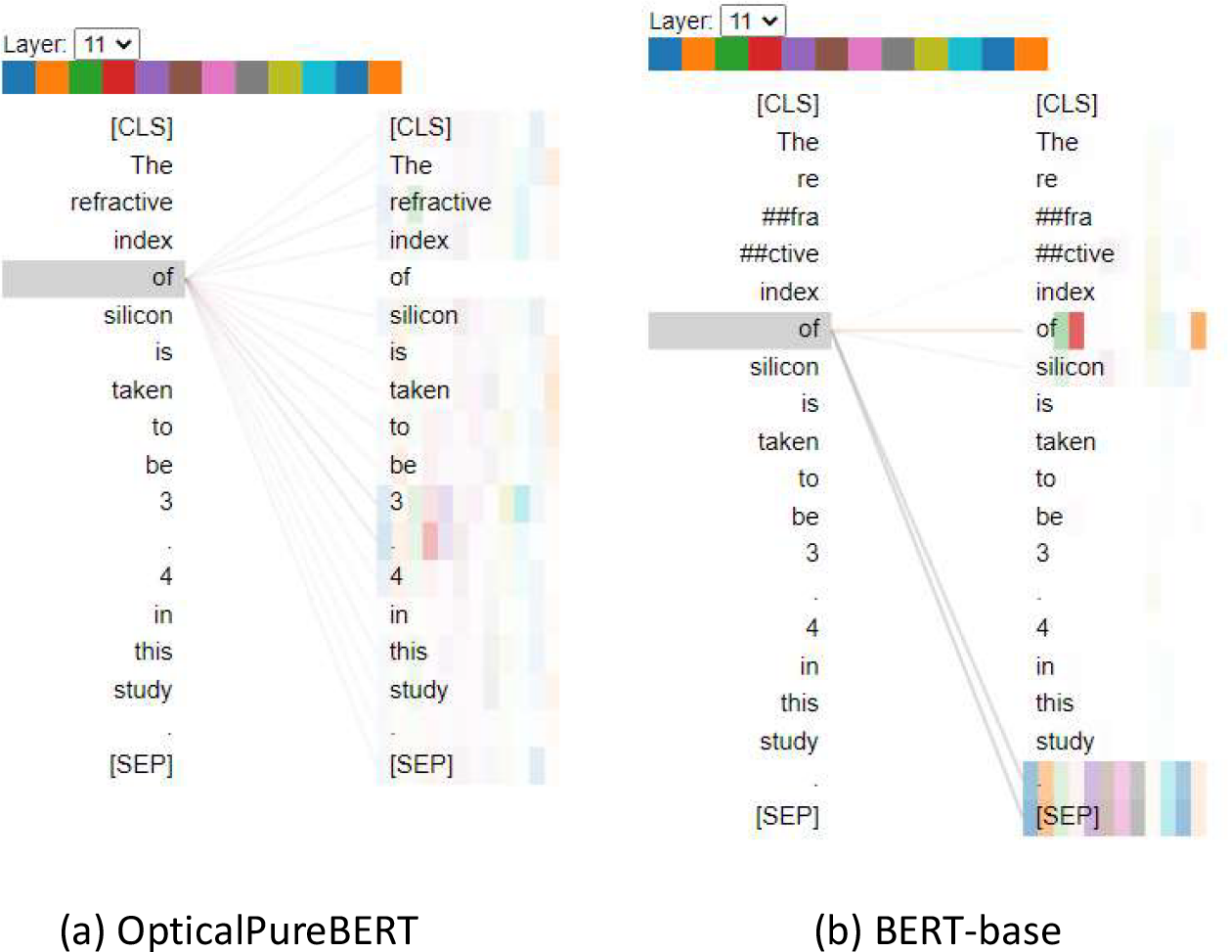
Visualization of self-attention weights in the OpticalPureBERT and BERT-base model architectures for the token “of” (highlighted) in Layer 11, i.e., the last layer of 12 layers (0–11). The layer contains 12 Heads, each of which is identifiable by color assignment, i.e., the color bar on the top of the figure.

**Figure 13:**
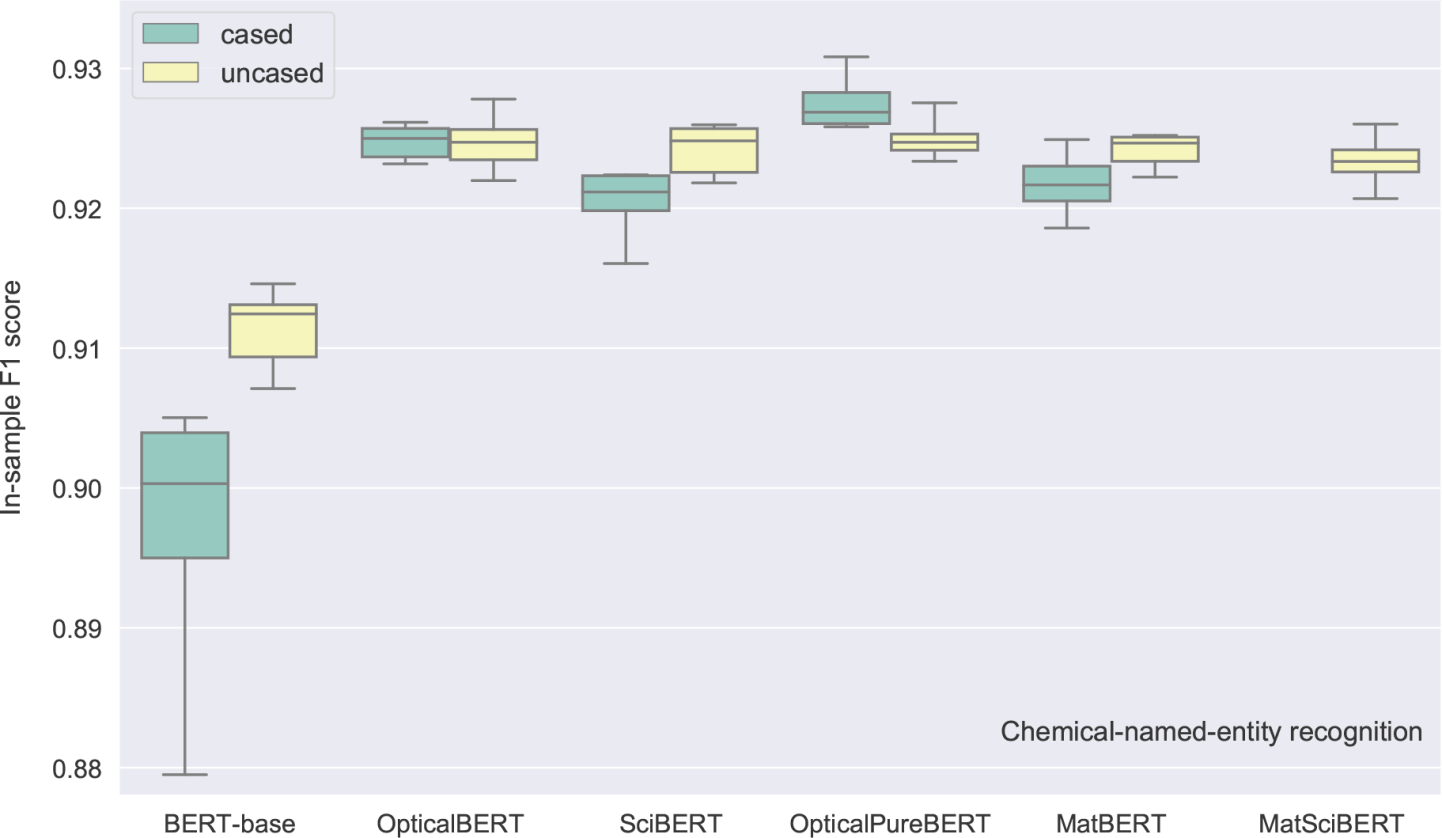
Box plots of the in-sample F1 scores for 11 fine-tuned BERT-based models when applied to the CNER development set. The statistical characteristics of each box pertain to the distribution of the F1 score of a given model that is evaluated against six hyperparameter sets.

**Figure 14:**
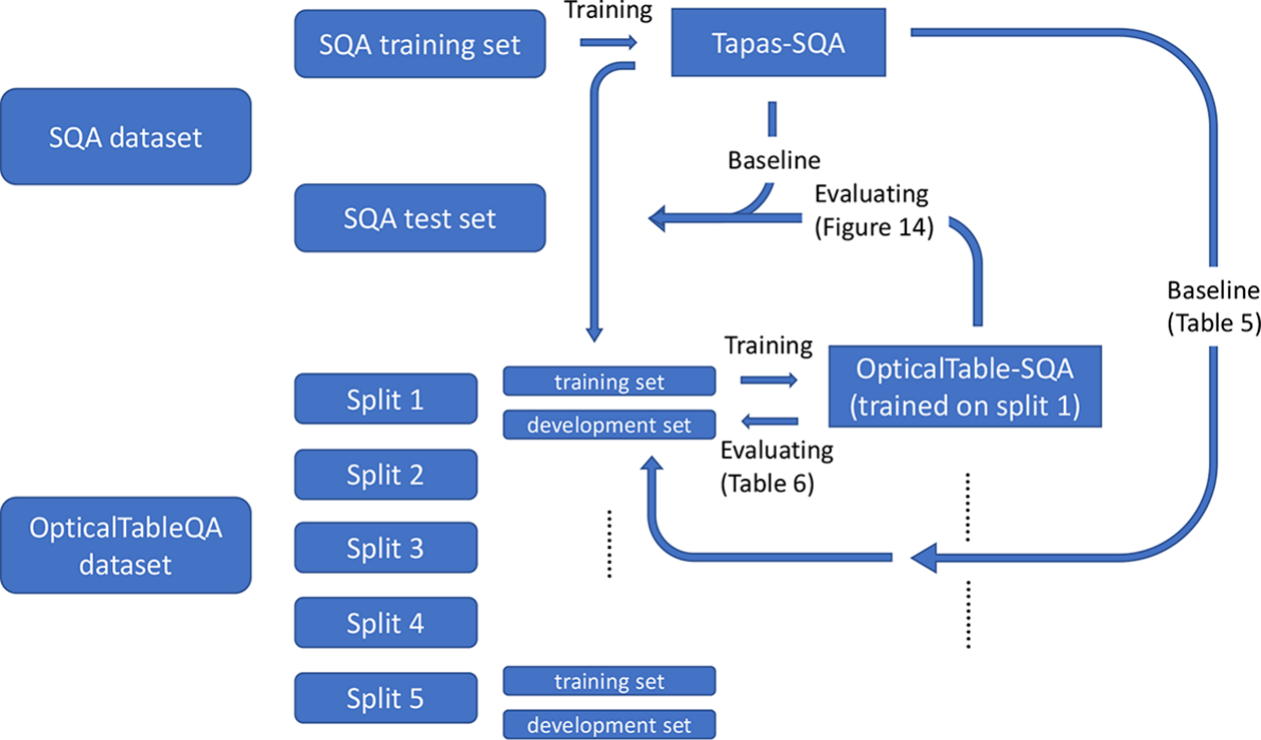
Schematic diagram showing the relationships between two data sets (SQA data set^41^ and OpticalTableQA data set) and two models (Tapas-SQA model^22^ and OpticalTable-SQA model). The SQA training set and SQA test set were preidentified by the data-set creator. The five splits of the OpticalTableQA data set were random and independent of each other.

**Figure 15:**
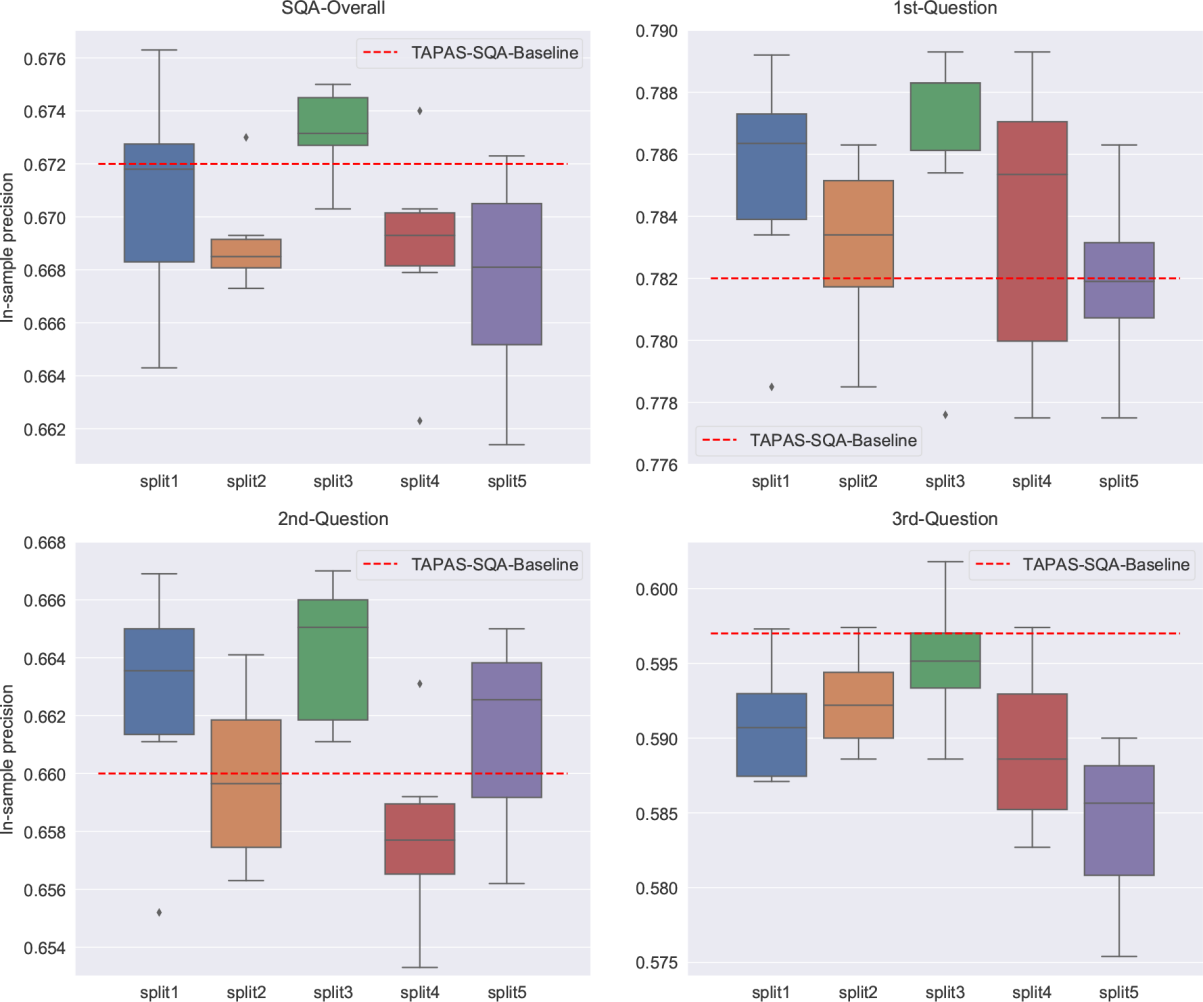
Box plots of the a) overall precision, b) first-question precision, c) second-question precision, and d) third-question precision, for five OpticalTable-SQA models that were created by fine-tuning the Tapas-SQA model using five random splits of the OpticalTableQA data set and hence applied to the SQA test set.^41^ Each box represents a distribution of the precision values of a model when refined against six {learning rate, batch size, epoch number} sets. The baseline precisions of the original Tapas-SQA model are shown via dotted lines.

All BERT-based models were fine-tuned to afford their specific functionality for abstract classification, question-answering, and CNER tasks. This fine-tuning procedure involved the optimization of three hyperparameters, i.e., the learning rate, batch size, and the number of epochs, in order to realize the best-performing model on its corresponding development set. Instead of only reporting the result of the model with the best {learning rate, batch size, epoch} set, we enumerated the highest precisions of the models for each {learning rate, batch size} set, i.e., the precision of {learning rate, batch size, the best number of epochs} set, and reported the distributions of these precisions in Figures 9–15; this provided a more comprehensive comparison between the models that were evaluated. As there were three candidates for tuning the learning rate (1*e*^–5^, 2*e*^–5^, and 5*e*^–5^) and two candidates for tuning the batch size (16, 32), each box in Figures 9–15 represents the distribution of six precisions for each model. While each box is bounded by the lower and upper quartiles of the precisions, whiskers extend to the furthest data point within the 4 * interquartile range. More extreme points are marked as outliers, in order to exclude some hyperparameters that may cause severe overfitting or gradient explosion, which make certain data points less meaningful.^42^

Once the hyperparameters of the models were optimized, each model was fine-tuned with five different random weight initializations of their prediction layers. The average value of the precision, recall, and F1 score of the five weight initializations of these models on the out-of-sample test sets, as well as their standard deviations, is reported in Tables 1, 3, and 4.

**Table 1:** Performance of Various Models When Applied to the Abstract-Classification Task Using the out-of-Sample Test Data Set of Abstracts about Optical Materials and Nonoptical Materials, Where Precision (%) Was Used as the Performance Metric^a^

model (cased)	precision	recall	F1 score
Prompt	73.33	62.68	67.59
LR	77.14	63.36	69.57
BERT-base	81.10 ± 0.41	65.19 ± 1.06	72.30 ± 0.80
SciBERT	81.08 ± 0.71	62.44 ± 1.56	70.55 ± 1.23
OpticalBERT	81.90 ± 0.20	63.05 ± 0.61	71.25 ± 0.45
OpticalPureBERT	81.85 ± 0.36	62.81 ± 0.73	71.08 ± 0.59
MatBERT	81.71 ± 0.52	62.44 ± 0.89	70.79 ± 0.73

model (uncased)	precision	recall	F1 score
Prompt	63.81	**84.09**	72.55
LR	74.92	61.07	67.29
BERT-base	81.50 ± 0.48	64.39 ± 0.68	71.94 ± 0.54
SciBERT	81.14 ± 0.52	61.22 ± 1.12	69.79 ± 0.88
OpticalBERT	81.38 ± 0.51	61.65 ± 0.58	70.15 ± 0.51
OpticalPureBERT	**82.18** ± 0.42	65.31 ± 0.92	**72.78** ± 0.72
MatBERT	81.33 ± 0.37	64.12 ± 0.97	71.71 ± 0.73
MatSciBERT	81.59 ± 0.35	61.57 ± 0.37	70.15 ± 0.32

a “LR” represents logistic regression. The “cased” and “uncased” tags that follow the type of dataset indicate whether the model is cased or uncased. “MatBERT” and “MatSciBERT” refer to our own versions of fine-tuned MatBERT and MatSciBERT models. The average values and nonzero standard deviations represent the overall performance of five random weight initializations of these models.

### Abstract Classification

We first considered the ability of language models to classify the abstract of a paper into topics about optical materials versus nonoptical materials using the in-sample development set.

The fine-tuned results of all BERT-based models for abstract classification are shown in Figure 9, which show that they achieved an in-sample validation precision of above 93.5% on average. Compared with the BERT-base models, all other models that were trained on science-related corpora showed an increasing capability to determine whether or not a paper focuses on optical-materials research based on its abstract. Among these models, the cased and uncased OpticalPureBERT models achieved the highest in-sample precision in this binary classification task, which reveals the positive effect of domain-specific pretraining on BERT-based models. Another noticeable finding is that the interquartile range of the in-sample precision obtained from different hyperparameters, i.e., the length of the box, is considerably lower for the OpticalBERT and OpticalPureBERT models. This indicates that the performance of our models is more stable upon tuning the hyperparameters, and it may reveal another benefit which is the enhancement of model stability for this downstream task when BERT-based models are pretrained with domain-specific data.

An obvious limitation of the in-sample development set is that it is not sufficiently general. As a result of our method of data set construction, the in-sample sets only contain papers that were believed to be, or not to be, about optical materials by excluding papers from journals whose names were hard to classify. To measure the general performance of our language models, we chose the models that delivered the highest precision on the development set as the best models. Thereby, Table 1 reports these results together with the precision of these best models on this task where it employed a manually built out-of-sample test set of 315 randomly sampled abstracts.

Table 1 shows that the performance of all models drops by ∼13% for all models when applied to the test set, relative to the precision metrics that were realized using the development set. This emphasizes the difficulty of training BERT-based models that deliver well when applied to this binary classification task using real-world examples and the necessity of building such an out-of-sample test data set to evaluate such models. Logistic regression (LR) was used as a baseline for evaluating the performance of these models on the test set, which took the Term frequency-Inverse Document Frequency (Tf-IDF) features as inputs. All of the BERT-based fine-tuned models outperformed logistic regression by ∼4%–7%, which demonstrates the superiority of deep-learning-based language models over traditional machine-learning approaches. Within a narrow range of precision scores, the BERT-based models that were pretrained on materials-science-related corpora, i.e., OpticalPureBERT, our fine-tuned MatBERT,^18^ and our fine-tuned MatSciBERT,^17^ either matched or beat the performance of the BERT-base model. However, the BERT-base models are characterized by high recall scores, which are possibly due to the model having seen more general text (i.e., nonscientific corpus). Among all BERT-based models, the uncased OpticalPureBERT model achieved the highest F1 score, which corroborates the argument that domain-specific BERT-based language models perform better in domain-specific tasks (Figure 9). Meanwhile, we also tried simple prompt learning on this task by using the BERT-base model. Prompt learning is a zero-shot method that does not require any training data.^32^ By properly constructing prompt templates and criteria, prompt learning has the potential to achieve promising performance when applied to a downstream task.^43^ Here, the prompt template was designed to be a “fill-mask” task by appending the sentence “The topic of this research is [MASK].”. at the end of the abstract. The prompt method employs the BERT-base model to generate a few predictions of words with different confidence scores at the position of “[MASK]” within the sentence concerned, for example, “chemistry”, “biology”, or “health”. We only considered the predicted words that achieved the highest five confidence scores, and we classified the abstract according to the following criteria:

1. If the top five predicted words contain any of the ‘negative’ keywords: ‘agriculture’, ‘health’, ‘education’, ‘geology’, or ‘biology’, then this abstract is classified as a **nonoptical-materials-related** abstract.
2. If the top five predicted words do not contain the keywords stated in criterion 1 but do contain any of the following ‘positive’ keywords: ‘physics’, ‘laser’, ‘telecommunications’, or ‘chemistry’, then this abstract is classified as an **optical-materials-related** abstract.
3. If the top five predicted words do not contain any of the keywords stated in criteria 1 or 2, then this abstract is classified as a **nonoptical-materials-related** abstract.

According to Table 1, the best precision achieved by prompt learning on the test set is 73.33%. This value is slightly lower than that of the logistic-regression-baseline model. A recall score of 84.09% is observed on the uncased prompt-learning approach, which is significantly higher than any other model. However, a closer look at the classification report reveals that the precision of this prompt learning on positive samples is only 55.22%, which suggests that nearly half of the papers that are identified as being related to optical-materials research are not in fact related to optical materials. As the recall score only considers the ability of the classifier to find all the positive samples, the precision score reflects the accuracy of the classifier to classify both the positive and the negative samples, and it might be more significant in measuring the performance of the models in this task. Although the precision of the prompt learning approach is much lower than those of the BERT-based models, it can be further improved by carefully tuning the three criteria above. These results also reveal the potential of using this zero-shot prompt-learning method on downstream tasks of the language model, where it would be especially valuable in circumstances where high-quality annotated data in the materials-science domain are rare and difficult to access.

The uncased OpticalPureBERT model was chosen to be the best option for this binary classification task, judging from the performance of all models on both the development set and the test set. We applied this classifier to all papers in our corpus, and the resulting numbers of successfully classified papers are shown in Table 2.

**Table 2:** Numbers and Compositions of Optical-Materials-Related or Nonoptical-Materials-Related Papers from Two Publishers That Were Successfully Classified Using the Fine-Tuned OpticalPureBERT Model

type/publisher	Elsevier	RSC	total
nonoptical-related	397,520, 72%	20,479, 75%	417,999, 72%
optical-related	154,536, 28%	6,987, 25%	161,523, 28%
total	552,056, 100%	27,466, 100%	579,522, 100%

Table 2 reveals that ∼72% of the papers in our corpus do not actually focus on research about optical materials. A similar trend has been observed in the corpus that was used to train MatSciBERT,^17^ where only ∼15.4% of papers were determined to be about materials science.^17^ The higher topic-related fraction of papers in our corpus (27.87%) suggests that ours is of superior quality to that corpus. It should be noted that the manual labeling of journals by their titles can still include irrelevant papers, and a paper that is determined to be nonoptical-materials-related can still contain useful information about optical properties within its main text. This classification task will also become especially important when one wishes to perform large-scale data-extraction tasks. To that end, filtering out irrelevant papers and only focusing on topic-related papers can save a lot of computing resources and time. Figure 10 displays individual counts of optical-materials-related papers selected by our abstract classification model for each year of publication, to illustrate the growing nature of the number of publications in the optical-materials domain.

### Question-Answering Tasks on Text

The performance of our language models, when applying the question-answering task to text, was assessed using two conventional evaluation metrics: the exact match (EM) score and the F1 score. The EM score of a single question-answering pair will either be 1 if the extracted answer matches 100% with the correct answer or 0 otherwise. However, the amount of text within the extracted answer that matches entirely with the correct answers (called the ‘gold answers’) very often lies between 0% and 100%. Therefore, another useful metric, recall, which represents the word-level fraction of the gold answer that is predicted correctly, is used to characterize this situation. For example, the EM score will be 0 and the recall will be 5/8 when the gold answer is “left-handed materials or media with negative refractive index” and the predicted answer is “media with negative refractive index”. The F1 score provides an overall consideration of the EM score and the recall score, and it can be calculated as
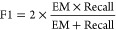
3

We first present an overview of the best F1 scores for BERT-based models that were applied to the in-sample development set where the performance across six {learning rate, batch size, epoch number} hyperparameter sets of each model was explored, with results being displayed in Figure 11.

The BERT-base and OpticalBERT models achieved the highest F1 scores when assessed using the development set of the SQuAD v1.1 data set. They outperformed the OpticalPureBERT and SciBERT models by, respectively, ∼0.6% and ∼1.5%. These results are totally reasonable because the latter two models were both trained from scratch on scientific papers only, and they had not seen general corpora such as Wikipedia. The outright highest score was achieved by the cased OpticalBERT model, which demonstrates that the performance of the original BERT-base model can be significantly enhanced by further pretraining it on domain-specific corpora and that this can also improve the accuracy of the question-answering task on general English data sets.

Table 3 reports the exact match score, recall, and F1 score for our best models that were obtained by applying them to two manually annotated out-of-sample test sets. To provide a more comprehensive comparison, we also calculated these scores for existing BERT-based models: SciBERT,^25^ our fine-tuned MatBERT,^18^ our fine-tuned MatSciBERT,^17^ and the original BERT-base model. Our ‘chemical-aware’ NLP-based tool, ChemDataExtractor,^4,5,8^ was used to construct the numerical test set; its performance in this data-extraction task is also stated in Table 3, so that it serves as a baseline for that test set when comparing the associated performances of various BERT-based models.

**Table 3:** Exact-Match, Recall, and F1 Scores of Various NLP-Based Models When Applied to Question-Answering Tasks on the out-of-Sample Test Sets^a^

arbitrary test set				numerical test set	
model (cased)	exact-match	recall	F1 score	model (cased)	precision
BERT-base	61.46	84.59	81.83	ChemDataExtractor	74.13
SciBERT	66.45	88.21	85.22	BERT-base	75.08
OpticalBERT	71.76	88.15	87.40	SciBERT	76.03
OpticalPureBERT	70.10	89.65	87.02	OpticalBERT	**87.70**
MatBERT	66.11	89.32	86.88	OpticalPureBERT	86.75
				MatBERT	84.22

arbitrary test set				numerical test set	
model (uncased)	exact-match	recall	F1 score	model (uncased)	precision
BERT-base	63.79	86.87	83.40	BERT-base	75.65 ± 1.40
SciBERT	67.44	88.83	86.48	SciBERT	84.22
OpticalBERT	69.10	87.47	86.47	OpticalBERT	86.44
OpticalPureBERT	**73.75**	89.84	**88.67**	OpticalPureBERT	87.38
MatBERT	68.10	90.27	86.98	MatBERT	86.12
MatSciBERT	69.10	**90.85**	88.32	MatSciBERT	85.17

a “MatBERT” and “MatSciBERT” refer to our own versions of fine-tuned MatBERT and MatSciBERT models. The average values and nonzero standard deviations represent the overall performance of five random weight initializations of these models. Zero standard deviations are omitted.

Table 3 shows that the performance of models pretrained from a science-related corpus significantly outperforms that of the BERT-base model when applied to the arbitrary test set, by ∼4%–12%. There are only two models that achieve an EM score of over 71%, the cased OpticalBERT and uncased OpticalPureBERT models, while the latter realizes the highest score (73.75%). The superior performance of the optical-materials-related BERT-based models on this domain-specific test set corroborates the aforementioned findings that the use of domain-specific corpora when pretraining a BERT-based is both significant and necessary. This improvement, brought by the two optical-related BERT-based models, is likely to arise due to more precise modeling of the word embeddings of the domain-specific tokens.

Meanwhile, we noticed that the most frequent incorrectness of the question-answering task when it was applied to these BERT-based models is the answer being incomplete, i.e., only some parts of the ‘gold answer’ were extracted, where the ‘gold answer’ often consists of several entities that are connected by commas, “and”, or “or”. This issue can be partially mitigated by considering not only the predicted answer with the highest confidence score (eq 2) but also the answers with the second- or third-highest confidence scores. Nevertheless, it also suggests the significance of including more such examples in the fine-tuning training data set.

We further evaluated the performance of these BERT-based models by applying them to our question-answering module while it is asked to query an out-of-sample numerical test set. Overall, BERT-based models that were pretrained from materials-science-based corpora, i.e., our fine-tuned MatBERT, MatSciBERT, OpticalPureBERT, and OpticalBERT models, drastically outperformed the BERT-base and the SciBERT models that were not pretrained on materials-science information. Overall, these model-performance results on this numerical data set are also a large improvement on those realized from the arbitrary test set on textual data. Precision of OpticalPureBERT and OpticalBERT (%) is slightly superior when compared with those of our fine-tuned MatBERT and MatSciBERT (∼85.5%), which suggests that the former models have seen more relationships of optical properties of materials during pretraining. The cased OpticalBERT model delivers the highest numerical precision (87.70%) when applied to the numerical question-answering test set. Compared with our baseline ‘chemical-aware’ data-mining approach, ChemDataExtractor,^4,5,8^ that employs traditional NLP, i.e., rule-based and semisupervised methods, to identify properties, BERT-based models extracted the correct property value better if several property values were presented in parallel within a sentence. For example, the refractive index of alumina (1.65) could be extracted from the sentence “The materials most widely used as AR coatings are dielectric materials: silica, titania, and alumina with refractive indices of 1.45, 2.3 and 1.65, respectively.” by answering the question, “What is the refractive index of alumina?”. The BERT-based models learn such bijective relationships between entities and numerical values during their pretraining and fine-tuning processes, while traditional NLP-based approaches tend to encode such relationships manually. The superior performance of the BERT-based models that were pretrained on materials-science corpora probably transpires from the fact that the model has seen similar types of relations during the masked pretraining process. The pretraining process in this study randomly masked 15% of the tokens and trained the BERT-based model to predict the identity of the masked tokens. In some cases, the property value was masked, e.g., “The refractive index of silicon is taken to be [MASK] in this study.”, where the identity of the masked token is “3.4”. A previous study has shown that the refractive index of silicon appears at least 1,200 times when this property is extracted from a corpus of ∼180k papers using ChemDataExtractor.^8^ Having seen such relationships of materials properties during this pretraining process, BERT-based models that have been pretrained on materials-science-related corpora are able to learn not only linguistic features but also a certain amount of domain-specific knowledge. To support this, Figure 12 visualizes the weights of 12 attention Heads of two BERT-based models, OpticalPureBERT and the original BERT-base, for the aforementioned sentence.

Each Head performs a linear transformation on tokens once they have been vectorized into token embeddings. The magnitude of the weight of each Head is represented in Figure 12 by the shade of color of the vertically stacked rectangles shown on the right side of each subdiagram. The overall magnitude of the weight between two tokens is given by the width of the lines that connect them, which reflects how much attention each token pays to other tokens within the text sequence. Figure 12 also illustrates the first notable difference between these two BERT-based models which is that the OpticalPureBERT model preserves the completeness of the word “refractive”, while BERT-base tokenizes it into “re”, “##fra”, and “##ctive”. This comes as one of the benefits of pretraining the OpticalPureBERT model on the domain-specific corpus and vocabulary. Second, if we focus on the token “of” in the sentence given in Figure 12, which is a critical preposition connecting “refractive index” and “silicon”, the OpticalPureBERT model notices that tokens ‘3’, ‘.’, and ‘4’ are crucial to that ‘of’, as indicated by the strong self-attention connections that are made between ‘of’ and ‘3’, ’.’, and ‘4’ in the bipartite graph of Figure 12(a). The relative strengths of the connections are denoted by the color and width of lines that are associated with these tokens. In contrast, the BERT-base model does not realize such significance between these tokens, judging from Figure 12(b). Although it should be noted that the relationships that exist between attention weights and model outputs are not statistically rigorous,^44^ this observation can still add some support to the notion that pretraining on domain-specific corpora permits a BERT-based model the possibility to learn domain-specific knowledge. This, in turn, also reveals the significance and superiority of using such a model to perform relationship extraction from documents at a later stage.

Although BERT-based question-answering modules achieve higher precision when extracting relationships from numerical data, they also have limitations that would need addressing before they could be used for relationship extraction. First, BERT-based models are less capable when dealing with long textual contents, e.g. paragraphs of more than 512 tokens.^19^ Also, relationship extraction cannot be achieved in any traditional or BERT-based NLP system that does not possess a sophisticated level of capability in CNER, which identifies chemical names within text.

### Chemical-Named-Entity Recognition (CNER)

The metrics used to characterize the performance of BERT-based models on a CNER task are microprecision, microrecall, and overall F1 scores. These metrics are perhaps best explained via an example. We consider the case of a validation set that contains 100 labeled chemical-named entities which have been extracted from 10 paragraphs of text; the model predicts there are 120 chemical names in these paragraphs, and out of those predictions, 70 predictions are correct. In this scenario, the microprecision is 70/120 = 58.3%, the microrecall is 70/100 = 70.0%, and the overall F1 score follows the same method of calculation from eq 3. Distributions of the F1 scores for eight BERT-based models, which have been evaluated across six {learning rate, batch size, epoch number} sets (same as Figures 9 and 11) using the in-sample development set, are shown in Figure 13.

Once again, BERT-based models that had been pretrained on scientific corpora show superior performance over the BERT-base model, while the cased OpticalPureBERT model delivered the highest performance for the F1 metric. Given that the interannotator agreements for CHEMDNER and Matscholar are 91%^39^ and 87%,^40^ respectively, one can see that our optical-related BERT-based models achieve close to human-level performance in general CNER tasks across both organic and inorganic domains. We further evaluated the efficacy of these optical-materials-related BERT models by checking their performance once fine-tuned for CNER tasks using the out-of-sample test set. Results are shown in Table 4 together with an entry that displays the comparable performance of the CNER function that is built into ChemDataExtractor v2.0^5,8^, when it is applied to this same test data set.

**Table 4:** Microprecision, Microrecall, and F1 Scores of Various NLP-Based Models When Applied to CNER Task on the out-of-Sample Test Data Set^a^

model (cased)	microprecision	microrecall	F1 score
ChemDataExtractor v2.0	81.60	71.17	76.03
BERT-base	81.17	83.20	82.17
SciBERT	79.01	**83.95**	81.41
OpticalBERT	80.90	80.70	80.80
OpticalPureBERT	**82.06**	83.71	**82.88**
MatBERT	76.71	75.94	76.32

model (uncased)	microprecision	microrecall	F1 score
ChemDataExtractor v2.0			
BERT-base	78.14	77.94	78.04
SciBERT	77.69	80.20	78.91
OpticalBERT	78.71	79.69	79.20
OpticalPureBERT	79.85	82.70	81.25
MatBERT	77.72	80.45	79.06
MatSciBERT	78.82	80.20	79.50

a “MatBERT” and “MatSciBERT” refer to our own versions of fine-tuned MatBERT and MatSciBERT models. The average values and nonzero standard deviations represent the overall performance of five random weight initializations of these models. Zero standard deviations are omitted.

Overall, all models perform worse on the out-of-sample test set than on the in-sample development set by ∼10%. This suggests that there is a quite large deviation between the textual nature of the chemical-named entities in the optical-materials domain and that of the biological domain, remembering that CHEMDNER was annotated from the text in the biological domain. All cased BERT-based models deliver higher F1 scores than those of their uncased models when they are applied to the test set, while this trend is not observed in the development set. This may indicate that more chemical-named entities in the optical-materials-science domain contain capitalized letters, so it is necessary to include the capitalization within chemical names when fine-tuning the BERT-based models and to use a cased model in real-world applications. Overall, the cased OpticalPureBERT model realizes the highest microprecision and F1 scores, which is natural given that this performance will be promoted by the domain-specific corpus that it employs in its pretraining stage. The cased SciBERT model delivers the highest microrecall. This is probably because this model benefits from being pretrained using a much larger training corpus (1.14 M papers). Another noticeable fact is that ChemDataExtractor v2.0^5,8^ achieves a promising microprecision on the test data set, either beating or matching the other fine-tuned BERT-based language models. This demonstrates the reliability and robustness of the traditional but ‘chemical-aware’ approach. Although the microrecall obtained from using ChemDataExtractor v2.0 does not reach that of the language models, its approach can be used as a reliable verification that the entities extracted by the language model is representative of real-world scenarios.

### Question-Answering Tasks on Tables

This section focuses on reporting several evaluation results of a new model, OpticalTable-SQA, which originates from the Tapas-SQA model,^22^ which has been further fine-tuned on our OpticalTableQA data set. The relationships between two data sets, i.e., the SQA data set^41^ and the OpticalTableQA data set, and two models, i.e., the original Tapas-SQA model^22^ and our OpticalTable-SQA model, and how these two models were evaluated are illustrated in Figure 14.

The size of our annotated OpticalTableQA data set is relatively small (it contains 4,534 question-answering pairs) compared to the data sets that were used in other evaluation tasks that have been reported in this study. So, we randomly split this data set into two, using an 80:20 ratio of data proportioning to create a training and development set, respectively; this splitting procedure was executed five times to minimize the contingency of just one random splitting. The Tapas-SQA^22,41^ model was initially applied to each development data subset for a given OpticalTableQA random split (1–5), to afford baseline precision values for each split, as shown in Table 5. These are baseline values because the Tapas-SQA model was designed and trained for parsing generic (i.e., nonscientific) tabular data, while it is applied here to data sets that contain optical-materials-related data from tables.

**Table 5:** Precision (%) of the Baseline Tapas-SQA Model of the Question-Answering Tasks That Were Applied to the Variably Split OpticalTableQA Development Sets^a^

split	what	which	overall
1	48.86	78.07	62.32
2	29.02	63.01	45.34
3	46.52	78.49	61.48
4	40.86	68.46	53.71
5	39.61	68.65	53.20

a Column “what” represents the precision of an answer to a “what”-question. Column “which” represents the precision of an answer to a “which”-question. Details about the question categories were described earlier, examples of which can be found in Figure 6.

Table 5 reveals that the overall extractive accuracy of the baseline Tapas-SQA model on tables that are contained within the optical-materials corpus is significantly lower than that which the Tapas-SQA model achieves when applied to tables of generic (i.e., nonscientific) information (78.2%).^22^ This lower accuracy mostly originates from the Tapas-SQA model not being able to understand symbols that represent optical properties in the table header. For example, the model does not realize that “λ_*absmax*_” refers to the maximum absorption wavelength when being asked, “What is the absorption maximum of the compound?”. The accuracy of a “which”-question is significantly higher than that of a “what”-question; this is because the model is able to identify the correct column within a table based just on the value provided by a “which”-question. For example, if we ask “Which compound was measured at a wavelength of 585 nm?” from the table shown in Figure 6 (top), the model may be able to locate the correct cell “Acetone” by only using keywords “compound” and “585”, rather than using the information contained in the table headers (i.e., “measurement wavelength”).

We addressed this problem by training the Tapas-SQA model on each of the five training sets that arose from the five split variants of the OpticalTableQA data set. The precision of the model on corresponding development sets once tuned on optical data is shown in Table 5. We called this newly tuned model OpticalTable-SQA and compared its results against the cognate performance of the baseline Tapas-SQA model (Table 5). We found that the data-extraction precision of the OpticalTable-SQA model delivers a significant improvement for all split data sets. Meanwhile, the precision of the OpticalTable-SQA model in answering the first question, when applied to the five development sets that were constructed by each random data set split, outperforms that of the baseline Tapas-SQA model on general Tables (78.2%). For data set splits 1, 3, and 5, the what-questions achieved a similar extractive precision to that of the which-questions. However, precision on the what-questions is lower than that of the which-questions by at least 6% for data set splits 2 and 4. These variations of model precision shown in Tables 5 and 6 emphasize the importance and necessity of evaluating the OpticalTable-SQA model on more than just one split.

**Table 6:** Precision (%) of the OpticalTable-SQA Model When Applied to the Variably Split Development Test Sets^a^

split	what	which	overall
1	89.14	90.98	90.00
2	80.10	86.54	83.19
3	87.87	89.04	88.42
4	77.22	87.82	82.16
5	81.67	85.31	83.37

a The column “what” and “which” have the same definition as described for Table 5.

We then sought to ensure that our fine-tuned model does not overfit on the training data and lose its generalizability on performing sequential question-answering tasks on tables that contain generic (i.e., nonscientific) text. Thereby, we investigated the performance of our OpticalTable-SQA model that was fine-tuned on five different random-split training sets (of the OpticalTableQA data set), when applied to the preidentified test set of the SQA data set,^41^ for which results are shown in Figure 15. It is observed that the overall precision of the OpticalTable-SQA models which were fine-tuned on splits 1 and 3 matches or beats that of the Tapas-SQA baseline precision, while that of the OpticalTable-SQA models which were fine-tuned on splits 2, 4, and 5 are lower than the baseline precision, as shown in Figure 15a. Question-answering precisions on the first question for all five OpticalTable-SQA models which were fine-tuned on five splits have been raised up, compared to that of the Tapas-SQA baseline, as shown in Figure 15b. For the second question (Figure 15c), OpticalTable-SQA models that were fined-tuned on splits 1, 2, 3, and 5 match or beat the baseline precision of the Tapas-SQA model. However, the precision of all OpticalTable-SQA models when applied to the third question of the SQA test set was suppressed. A clear trend in suppressed precision of the OpticalTable-SQA model with an increase in the question order is observed, when compared against the cognate performance of the Tapas-SQA baseline model. This is due to the fact that our OpticalTableQA data set contains purely the first-order question. By fine-tuning the Tapas-SQA model on a data set containing a larger fraction of first questions, the model precision for higher-order questions will be sacrificed, but the performance on the first and second questions gains certain improvements. This also confirms that our OpticalTableQA data set is of high quality, on which the fine-tuned model outperforms the previous state-of-the-art Tapas-SQA model on the first and second questions. Overall, evaluation results of the OpticalTable-SQA model, when applied to the development sets of OpticalTableQA data set and the preidentified test set of the SQA data set,^41^ demonstrate that Tapas-SQA models, which are fine-tuned on our OpticalTableQA data set, deliver a large enhancement in model precision of question-answering tasks on tables within the optical-materials-science domain, while they also preserve promising generalizability of question-answering tasks on tables containing generic (i.e., nonscientific) information.

**Figure 16:**
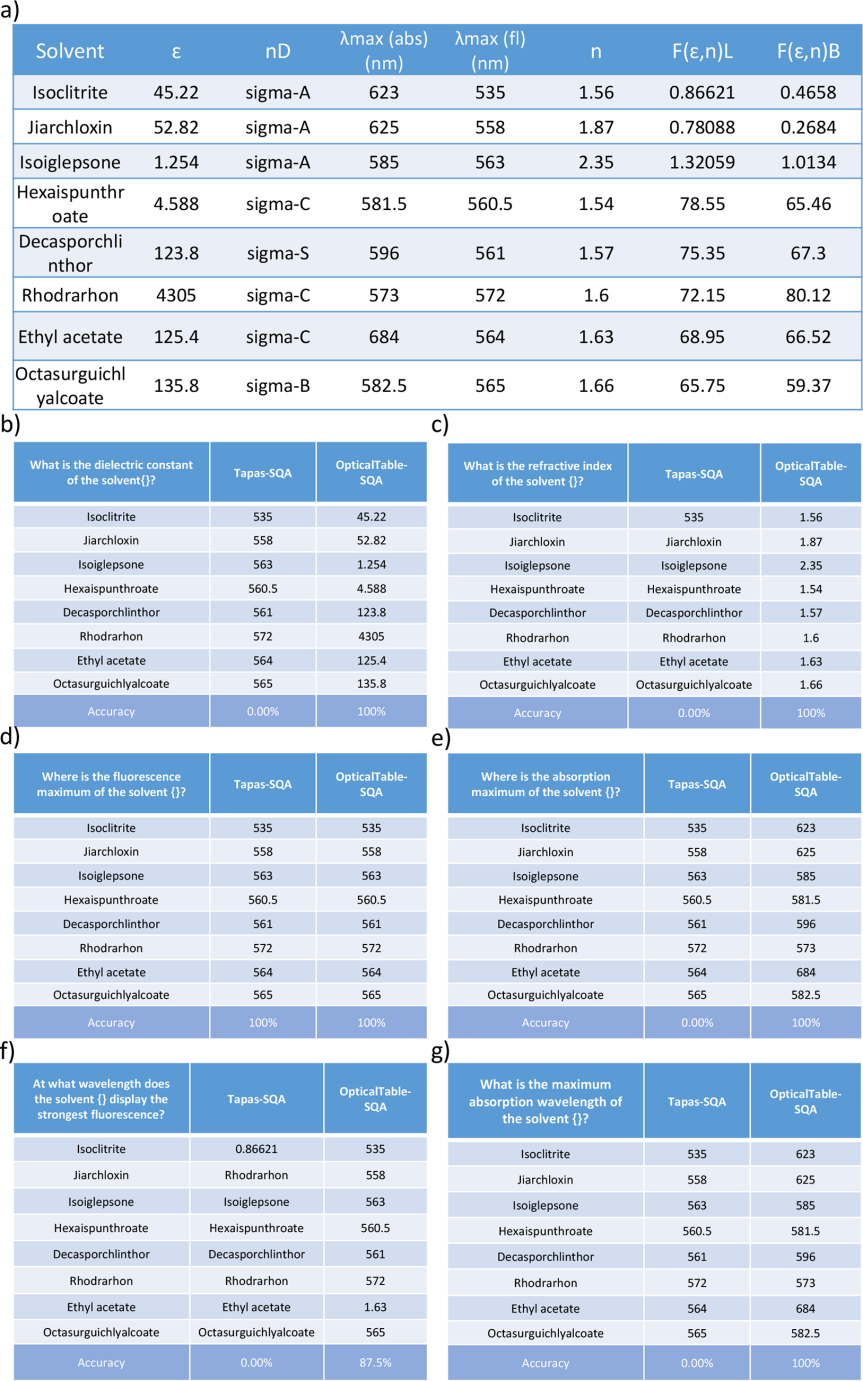
(a) The “fake” table used in the case study of the OpticalTable-SQA model. (b) Questions asking about the dielectric constant and (c) refractive index and their corresponding answers. (d) Questions asking about the wavelength fluorescence maximum and (e) absorption maximum and their corresponding answers. (f) Questions asking about the wavelength fluorescence maximum and (g) absorption maximum with different phrases and their corresponding answers. The question shown in the top-left cell of each table in Figure 16(b-g) is asked for each of the solvents in the “fake” table of Figure 16(a). The brace at the end of the question shows the position at which a solvent name will be inserted.

At last, we fine-tuned the Tapas-SQA model by using the entire OpticalTableQA data set, which generates a complete and final OpticalTable-SQA model. This model is used in a case study to demonstrate its level of extractive capability on tables about optical materials. A synthetic table was created for this case study, as shown in Figure 16a. Its column header contains four optical properties: dielectric constant (ϵ), refractive index (n), absorption maximum (λmax (abs)), and fluorescence maximum (λmax (fl)). Three other column headers, nD, F(ϵ,n)L, and F(ϵ,n)B were also appended to the table as “distracting” terms. The first column contains eight “fake” solvents, whose names were created by using a Molecular name generator (https://www.fantasynamegenerators.com/molecule-names.php) with some patterns; these names do not exist in the real world. The use of this fake table also prevented information leakage of real chemical names from the training set. Figure 16(b)-(g) shows the question of this case study and the answers that were realized using either the baseline Tapas-SQA model or our final OpticalTable-SQA model.

We first employed two individual questions that ask about the dielectric constant and the refractive index of these solvents (Figure 16b and c). The baseline Tapas-SQA model fails to understand the optical property represented by a single character (ϵ, n) in Figure 16a. This is reasonable since the SQA data set does not contain any training examples from the scientific text along the optical-materials domain. The OpticalTable-SQA model achieves a precision of 100% on these two questions. In Figure 16d and e, we then asked two individual questions about the content of the fake table using phrases that were not in our OpticalTableQA data set, in order to investigate whether or not our model could distinguish two optical properties that are represented by very similarly looking symbols. Although the Tapas-SQA model correctly answered the question about the fluorescence maximum, it failed to distinguish “λmax (abs)” and “λmax (fl)”, as it provided the same answers for these two questions. Meanwhile, the OpticalTable-SQA model showed its ability to distinguish between minimal differences in property specifiers. Two additional questions (Figure 16f and g) were constructed such that we changed the phrase of the question but kept it with the same meaning (as Figure 16d and e) in order to demonstrate the robustness of the OpticalTable-SQA model, while these two questions also did not present in our OpticalTableQA data set. The results (Figure 16f and g) show that the OpticalTable-SQA still delivers satisfactory performance when the phrase of the question has been slightly changed (Figure 16f) or has been significantly changed (Figure 16g), while the Tapas-SQA model fails to give the correct answer in both cases.

**Figure 17:**
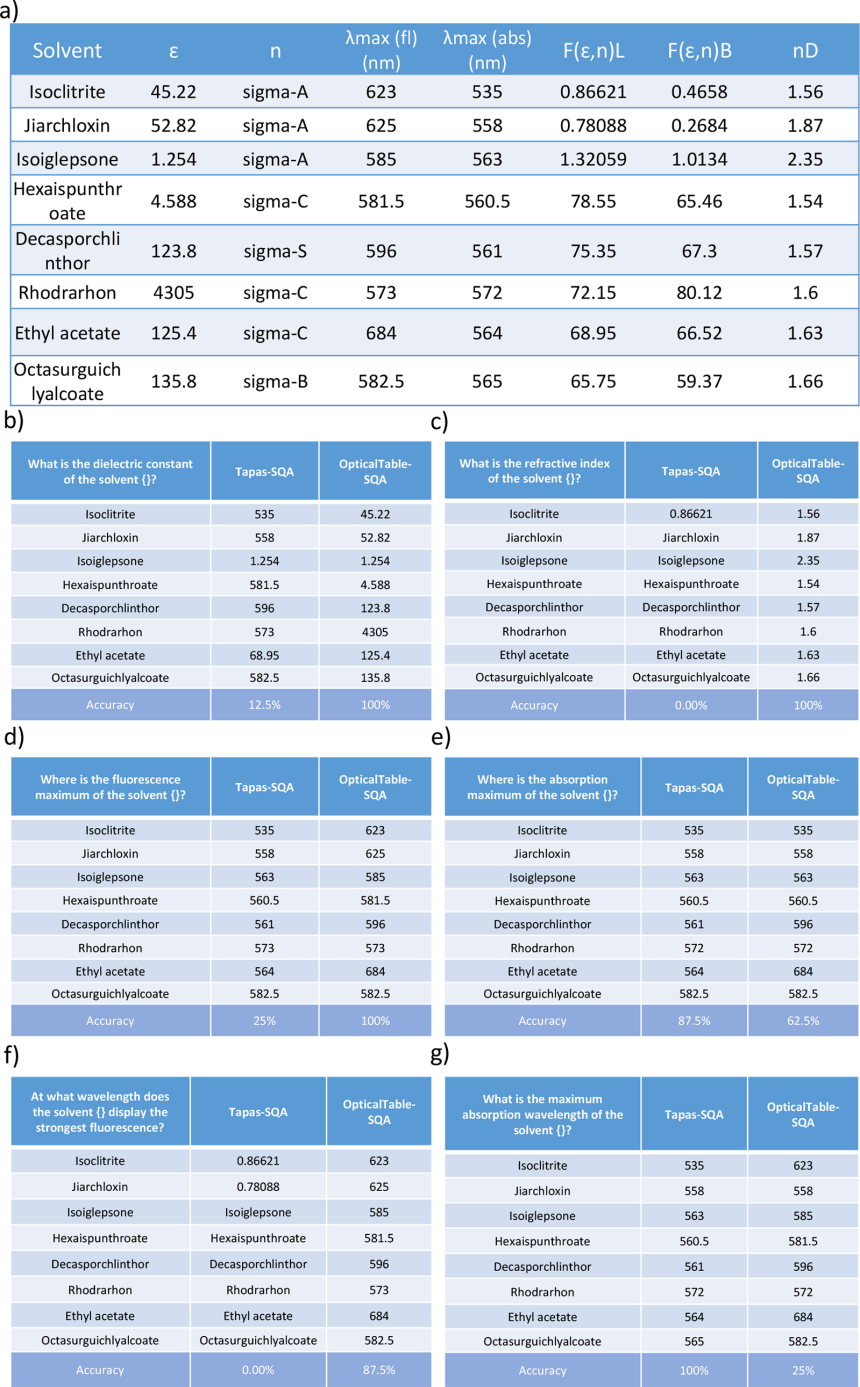
(a) The modified “fake” table used in the case study of the OpticalTable-SQA model. (b) Questions asking about the dielectric constant and (c) refractive index and their corresponding answers. (d) Questions asking about the wavelength fluorescence maximum and (e) absorption maximum and their corresponding answers. (f) Questions asking about the wavelength fluorescence maximum and (g) absorption maximum with different phrases and their corresponding answers. The question shown in the top-left cell of each table in Figure 17(b-g) is asked for each of the solvents in the “fake” table of Figure 17(a). The brace at the end of the question shows the position at which a solvent name will be inserted.

Tapas and co-workers have shown that embeddings of the row index and column index play the most important role in the performance of the original Tapas-SQA model.^22^ Thus, the order of columns might have a significant effect on its extractive power. We tested this hypothesis by swapping the column header “n” with “nD” and “λmax (abs)” with “λmax (fl)”, while the column content was left unchanged, and we moved the column “nD” to the end of the table. The same six questions were then asked. Their answers, together with the modified table, are shown in Figure 17. Even though “n” and “nD” had been switched, our OpticalTable-SQA model still correctly identified that the correct column which represents the refractive index is the column containing the column header “nD” instead of “n”. This result reveals one of the key differences between these Tapas-based language-model approaches for data extraction from tables and conventional rule-based table data-extraction using tools such as TableDataExtractor;^5^ i.e., the data-extraction process pertaining to the language model relies not only on the property specifier but also on a large variety of features such as the table structure, the cell content, and the size relationship between numbers. In the second example (Figure 17c), the correctly identified column for the refractive index contains pure numbers, while the “distracting” column contains general English words. Our OpticalTable-SQA model demonstrates a certain capability in using the cell content and the table structure to extract the correct answer. However, the extractive precision of the OpticalTable-SQA model of answering the maximum absorption wavelength is suppressed when “λmax (abs)” and “λmax (fl)” are switched (Figure 17e and g). This is possibly due to the large similarity between both the property specifiers and content of cells, and it is very rare and not logical that “λmax (fl)” appears in preceding “λmax (abs)” in a given table in our OpticalTableQA data set. The performance of the OpticalTable-SQA model can be further improved by introducing data augmentation to the OpticalTableQA data set, i.e., creating “fake” tables whose column headers are switched, which is analogous to adding rotated pictures to the training data set in an image-classification task.

Although the OpticalTable-SQA model has demonstrated superior data-extractive precision and robustness when applied to QA tasks on tabular data about optical materials, it also preserves several limitations of the original Tapas-SQA model. First, this original model is an uncased model such that it converts all capitalized words into words with only lowercase letters during pretraining and fine-tuning processes. This limits the expressive ability of the Tapas-SQA model even when it has been tuned using the OpticalTableQA data set, as most of the symbols of chemical elements and physical or chemical properties are capitalized; for example, it is not possible for an uncased Tapas-based model to distinguish between “eg” and “E_g_” or between “as” and “As”, where these words may represent the band gap or an elemental constituent of material, respectively. Second, the vocabulary and tokenizer of the Tapas-SQA model were trained from corpora of generic tabular data (e.g., WikiTable). The model performance could be further improved by using a domain-specific vocabulary and tokenizer when it is applied to a materials-science domain. For example, the word “fluorescence” is tokenized into [“flu”, “##orescence”] by the default Tapas tokenizer (and vocabulary), while the tokenizer of the OpticalPureBERT model will yield a result of [“fluorescence”]. Preserving the completeness of domain-specific tokens during tokenization will benefit the performance of many downstream tasks.^30^ Also, as was illustrated in Figure 7, the OpticalTableQA data set that was used to create the OpticalTable-SQA model displays a substantial bias toward two optical properties: the refractive index and dielectric constant. Thus, the performance of our OpticalTable-SQA model on other optical properties will be naturally less satisfactory. Third, the real-world application of our OpticalTable-SQA model also suffers from some systematic limitations. For example, the length of a table that it can parse is limited to 512 tokens once the table has been tokenized, which is approximately equivalent to a table of 15 rows and six columns; longer tables have to be truncated. Moreover, the Tapas model allows discrete reasoning over the table, such as summing numbers or counting cells.^22^ We intend to equip the OpticalTable-SQA model with such aggregation-operator functionalities as the training data set grows. Overall, our OpticalTable-SQA model could be further improved both in its data-extractive precision and its generalizability to generic text by pretraining it on a cased domain-specific corpus and fine-tuning it on a data set of tables about optical materials which has a larger size.

## Conclusion

In this article, we have introduced three new language models: OpticalBERT, OpticalPureBERT, and OpticalTable-SQA. The former two models were pretrained on an optical-materials-based corpus; they differ by virtue of the fact that the OpticalBERT model was trained from the initial weights of the BERT-base model, while the OpticalPureBERT model was trained from scratch. We evaluated the performance of these two models on three downstream tasks: abstract classification, extractive question answering, and chemical-named-entity recognition (CNER). For each task, we built a manually annotated out-of-sample test data set using a corpus from the optical-materials-science domain. These optical-related BERT-based models demonstrated superior performance over the original BERT-base model^15^ and the SciBERT model^25^ on all three downstream tasks. The OpticalTable-SQA model provides a means to extract information from tabular data of documents about optical materials, whereby it has been tailored for use in this scientific domain. The model was created by fine-tuning the Tapas-SQA model^22^ using the OpticalTableQA data set, which was curated specifically for this study. The OpticalTableQA data set contains 4,534 manually annotated question-answering pairs from a corpus of tables whose content pertains to the optical-materials-science domain. Our OpticalTable-SQA model significantly outperforms the Tapas-SQA model on optical-materials-related tables, while it preserves or even beats the model performance of Tapas-SQA on tables that display generic content. All of these new language models have been made available to the optical-materials-science community via this publication.

Our BERT-based models employed the BERT-base architecture as its standing basis. This choice was made owing to the already computationally intense nature of this work. As computing resources continue to become more powerful, future work could consider employing models such as BERT-large for the baseline architecture in the development of more sophisticated BERT-based models that serve the optical-materials-research community. The greater number of layers and larger parameter space of the BERT-large model, compared with the architecture of the BERT-base model, could bring a significant improvement in model performance on all tasks. The performance of question-answering tasks on tabular data could also be improved by further enriching the variety of optical properties in the OpticalTableQA data set, since this will bring enhanced robustness to our OpticalTable-SQA model. Overall, our new models offer relationship-extraction capabilities for text-mining that can be used to build bespoke materials databases about optical properties whose quality is promising; thereby, our new tools will help to accelerate information extraction from the optical-materials-science domain.

## Data and Software Availability

The fine-tuned language models for the abstract classification, question-answering task for text, chemical-named-entity recognition, and question-answering task for tables are available at https://huggingface.co/opticalmaterials. Corresponding out-of-sample test data sets can be found at https://huggingface.co/datasets/opticalmaterials/test_datasets. The OpticalTableQA data set is available free of charge at https://huggingface.co/datasets/opticalmaterials/OpticalTableQA.

## Supporting Information Available

Implementation details of the pretraining process of BERT-based models, Evaluation details of the abstract classification development data set, Evaluation details of the development data set of the question-answering task for text, Evaluation details of the chemical-named-entity recognition development data set, Evaluation details of different splits of the OpticalTableQA data set, and Out-of-sample test data sets of four tasks (ZIP).

## References

[ref1] Garcia de Abajo F. J. Engineering Materials with Extreme Optical Properties. In Proceedings of the Photonic Metamaterials: From Random to Periodic, TuA2; Optical Society of America, 2006.

[ref2] Higashihara T.; Ueda M. Recent Progress in High Refractive Index Polymers. Macromolecules 2015, 48 (7), 1915–1929. 10.1021/ma502569r.

[ref3] Panigrahi S.; Gioux S. Machine learning approach for rapid and accurate estimation of optical properties using spatial frequency domain imaging. J. Biomed. Opt. 2019, 24 (7), 1–6. 10.1117/1.JBO.24.7.071606. PMC6995874 30550050

[ref4] Swain M. C.; Cole J. M. ChemDataExtractor: A Toolkit for Automated Extraction of Chemical Information from the Scientific Literature. J. Chem. Inf. Model. 2016, 56 (10), 1894–1904. 10.1021/acs.jcim.6b00207. 27669338

[ref5] Mavračić J.; Court C. J.; Isazawa T.; Elliott S. R.; Cole J. M. ChemDataExtractor 2.0: Autopopulated Ontologies for Materials Science. J. Chem. Inf. Model. 2021, 61 (9), 4280–4289. 10.1021/acs.jcim.1c00446. 34529432

[ref6] Huang S.; Cole J. M. A database of battery materials auto-generated using ChemDataExtractor. Sci. Data 2020, 7 (1), 260 10.1038/s41597-020-00602-2. 32764659 PMC7411033

[ref7] Court C. J.; Cole J. M. Auto-generated materials database of Curie and Néel temperatures via semi-supervised relationship extraction. Sci. Data 2018, 5, 180111 10.1038/sdata.2018.111. 29917013 PMC6007086

[ref8] Zhao J.; Cole J. M. A database of refractive indices and dielectric constants auto-generated using ChemDataExtractor. Sci. Data 2022, 9 (1), 192 10.1038/s41597-022-01295-5. 35504964 PMC9065060

[ref9] Beard E. J.; Sivaraman G.; Vázquez-Mayagoitia Á.; Vishwanath V.; Cole J. M. Comparative dataset of experimental and computational attributes of UV/vis absorption spectra. Sci. Data 2019, 6 (1), 307 10.1038/s41597-019-0306-0. 31804487 PMC6895184

[ref10] Beard E. J.; Cole J. M. Perovskite- and Dye-Sensitized Solar-Cell Device Databases Auto-generated Using ChemDataExtractor. Sci. Data 2022, 9, 329 10.1038/s41597-022-01355-w. 35715446 PMC9205998

[ref11] Dong Q.; Cole J. M. Auto-generated database of semiconductor band gaps using ChemDataExtractor. Scientific Data 2022, 9 (1), 193 10.1038/s41597-022-01294-6. 35504897 PMC9065101

[ref12] Kumar P.; Kabra S.; Cole J. M. Auto-generating databases of Yield Strength and Grain Size using ChemDataExtractor. Sci. Data 2022, 9 (1), 292 10.1038/s41597-022-01301-w.

[ref13] Peters M. E.; Neumann M.; Iyyer M.; Gardner M.; Clark C.; Lee K.; Zettlemoyer L. Deep Contextualized Word Representations. In Proceedings of the 2018 Conference of the North American Chapter of the Association for Computational Linguistics: Human Language Technologies, Vol. 1 (Long Papers), 2227–2237; Association for Computational Linguistics, New Orleans, LA, 2018; 10.18653/v1/N18-1202.

[ref14] Brown T. B.; Mann B.; Ryder N.; Subbiah M.; Kaplan J.; Dhariwal P.; Neelakantan A.; Shyam P.; Sastry G.; Askell A.; Agarwal S.; Herbert-Voss A.; Krueger G.; Henighan T.; Child R.; Ramesh A.; Ziegler D. M.; Wu J.; Winter C.; Hesse C.; Chen M.; Sigler E.; Litwin M.; Gray S.; Chess B.; Clark J.; Berner C.; McCandlish S.; Radford A.; Sutskever I.; Amodei D. Language Models are Few-Shot Learners. 2020, 2005.14165. ArXiv2005.14165. https://arxiv.org/abs/2005.14165 (accessed 2023-03-06).

[ref15] Devlin J.; Chang M.; Lee K.; Toutanova K. BERT: Pre-training of Deep Bidirectional Transformers for Language Understanding. 2018, 1810.04805. ArXiv1810.04805. https://arxiv.org/abs/1810.04805 (accessed 2023-03-06).

[ref16] Lee J.; Yoon W.; Kim S.; Kim D.; Kim S.; So C. H.; Kang J. BioBERT: a pre-trained biomedical language representation model for biomedical text mining. 2019, 1901.08746. ArXiv1901.08746. https://arxiv.org/abs/1901.08746 (accessed 2023-03-06). 10.1093/bioinformatics/btz682 PMC7703786 31501885

[ref17] Gupta T.; Zaki M.; Krishnan N. M. A.; Mausam. MatSciBERT: A Materials Domain Language Model for Text Mining and Information Extraction. 2021, 2109.15290. ArXiv2109.15290. https://arxiv.org/abs/2109.15290 (accessed 2023-03-06).

[ref18] Trewartha A.; Walker N.; Huo H.; Lee S.; Cruse K.; Dagdelen J.; Dunn A.; Persson K. A.; Ceder G.; Jain A. Quantifying the advantage of domain-specific pre-training on named entity recognition tasks in materials science. Patterns 2022, 3 (4), 100488 10.1016/j.patter.2022.100488. 35465225 PMC9024010

[ref19] Huang S.; Cole J. M. BatteryBERT: A Pretrained Language Model for Battery Database Enhancement. J. Chem. Inf. Model 2022, 62, 6365 10.1021/acs.jcim.2c00035. 35533012 PMC9795558

[ref20] National Science and Technology Council. Materials Genome Initiative for Global Competitiveness; Executive Office of the President, National Science and Technology Council, 2011.

[ref21] Vakulenko S.; Savenkov V. TableQA: Question Answering on Tabular Data. 2017, 1705.06504. ArXiv1705.06504. https://arxiv.org/abs/1705.06504 (accessed 2023-03-06).

[ref22] Herzig J.; Nowak P. K.; Müller T.; Piccinno F.; Eisenschlos J. M. Tapas: Weakly Supervised Table Parsing via Pre-training. In Proceedings of the 58th Annual Meeting of the Association for Computational Linguistics (Vol. 1: Long Papers); Seattle, Washington, United States, 2020; 10.18653/v1/2020.acl-main.398.

[ref23] Chemmengath S. A.; Kumar V.; Bharadwaj S.; Sen J.; Canim M.; Chakrabarti S.; Gliozzo A.; Sankaranarayanan K. Topic Transferable Table Question Answering. 2021, 2109.07377. ArXiv2109.07377. https://arxiv.org/abs/2109.07377 (accessed 2023-03-06).

[ref24] Vaswani A.; Shazeer N.; Parmar N.; Uszkoreit J.; Jones L.; Gomez A. N.; Kaiser L.; Polosukhin I. Attention Is All You Need. 2017, 1706.03762. ArXiv1706.03762. https://arxiv.org/abs/1706.03762 (accessed 2023-03-06).

[ref25] Beltagy I.; Cohan A.; Lo K. SciBERT: Pretrained Contextualized Embeddings for Scientific Text. 2019, 1903.10676. ArXiv1903.10676. https://arxiv.org/abs/1903.10676 (accessed 2023-03-06).

[ref26] Wolf T.; Debut L.; Sanh V.; Chaumond J.; Delangue C.; Moi A.; Cistac P.; Rault T.; Louf R.; Funtowicz M.; Brew J. HuggingFace’s Transformers: State-of-the-art Natural Language Processing. 2019, 1910.03771. ArXiv1910.03771. https://arxiv.org/abs/1910.03771 (accessed 2023-03-06).

[ref27] Wu Y.; Schuster M.; Chen Z.; Le Q. V.; Norouzi M.; Macherey W.; Krikun M.; Cao Y.; Gao Q.; Macherey K.; Klingner J.; Shah A.; Johnson M.; Liu X.; Kaiser L.; Gouws S.; Kato Y.; Kudo T.; Kazawa H.; Stevens K.; Kurian G.; Patil N.; Wang W.; Young C.; Smith J.; Riesa J.; Rudnick A.; Vinyals O.; Corrado G.; Hughes M.; Dean J. Google’s Neural Machine Translation System: Bridging the Gap between Human and Machine Translation. 2016, 1609.08144. ArXiv1609.08144. https://arxiv.org/abs/1609.08144 (accessed 2023-03-06).

[ref28] Venn M.A J. On the diagrammatic and mechanical representation of propositions and reasonings. London, Edinburgh, and Dublin Philosophical Magazine and Journal of Science 1880, 10 (59), 1–18. 10.1080/14786448008626877.

[ref29] Ács J. Exploring BERT’s Vocabulary. http://juditacs.github.io/2019/02/19/bert-tokenization-stats.html (accessed 2022-05-30).

[ref30] Rust P.; Pfeiffer J.; Vulic I.; Ruder S.; Gurevych I. How Good is Your Tokenizer? On the Monolingual Performance of Multilingual Language Models. 2020, 2012.15613. ArXiv2012.15613. https://arxiv.org/abs/2012.15613 (accessed 2023-03-06).

[ref31] Paszke A.; Gross S.; Chintala S.; Chanan G.; Yang E.; DeVito Z.; Lin Z.; Desmaison A.; Antiga L.; Lerer A. Automatic Differentiation in PyTorch. In NIPS 2017 Workshop on Autodiff ; 2017.

[ref32] Liu P.; Yuan W.; Fu J.; Jiang Z.; Hayashi H.; Neubig G. Pre-train, Prompt, and Predict: A Systematic Survey of Prompting Methods in Natural Language Processing. 2021, 2107.13586. ArXiv2107.13586. https://arxiv.org/abs/2107.13586 (accessed 2023-03-06).

[ref33] Rajpurkar P.; Zhang J.; Lopyrev K.; Liang P. SQuAD: 100, 000+ Questions for Machine Comprehension of Text. 2016, /1606.05250. ArXiv1606.05250. https://arxiv.org/abs/1606.05250 (accessed 2023-03-06).

[ref34] Wiese G.; Weissenborn D.; Neves M. L. Neural Domain Adaptation for Biomedical Question Answering. 2017, 1706.03610. ArXiv1706.03610. https://arxiv.org/abs/1706.03610 (accessed 2023-03-06).

[ref35] Giorgi J. M.; Bader G. D. Transfer learning for biomedical named entity recognition with neural networks. Bioinformatics 2018, 34 (23), 4087–4094. 10.1093/bioinformatics/bty449. 29868832 PMC6247938

[ref36] Wang X.; Zhang Y.; Ren X.; Zhang Y.; Zitnik M.; Shang J.; Langlotz C. P.; Han J. Cross-type Biomedical Named Entity Recognition with Deep Multi-Task Learning. 2018, 1801.09851. ArXiv1801.09851. https://arxiv.org/abs/1801.09851 (accessed 2023-03-06). 10.1093/bioinformatics/bty869 30307536

[ref37] Isazawa T.; Cole J. M. Single Model for Organic and Inorganic Chemical Named Entity Recognition in ChemDataExtractor. J. Chem. Inf. Model 2022, 62 (5), 1207–1213. 10.1021/acs.jcim.1c01199. 35199519 PMC9049593

[ref38] Sang E. F. T. K.; Buchholz S. Introduction to the CoNLL-2000 Shared Task: Chunking. 2000, cs.CL/0009008. arXiv:cs/0009008. https://arxiv.org/abs/cs/0009008 (accessed 2023-03-06).

[ref39] Krallinger M.; Rabal O.; Leitner F.; Vazquez M.; Salgado D.; Lu Z.; Leaman R.; Lu Y.; Ji D.; Lowe D. M.; Sayle R. A.; Batista-Navarro R. T.; Rak R.; Huber T.; Rocktäschel T.; Matos S.; Campos D.; Tang B.; Xu H.; Munkhdalai T.; Ryu K. H.; Ramanan S. V.; Nathan S.; Žitnik S.; Bajec M.; Weber L.; Irmer M.; Akhondi S. A.; Kors J. A.; Xu S.; An X.; Sikdar U. K.; Ekbal A.; Yoshioka M.; Dieb T. M.; Choi M.; Verspoor K.; Khabsa M.; Giles C. L.; Liu H.; Ravikumar K. E.; Lamurias A.; Couto F. M.; Dai H.-J.; Tsai R. T.-H.; Ata C.; Can T.; Usié A.; Alves R.; Segura-Bedmar I.; Martínez P.; Oyarzabal J.; Valencia A. The CHEMDNER corpus of chemicals and drugs and its annotation principles. J. Cheminformatics 2015, 7 (1), S2 10.1186/1758-2946-7-S1-S2. PMC4331692 25810773

[ref40] Weston L.; Tshitoyan V.; Dagdelen J.; Kononova O.; Trewartha A.; Persson K. A.; Ceder G.; Jain A. Named Entity Recognition and Normalization Applied to Large-Scale Information Extraction from the Materials Science Literature. J. Chem. Inf. Model 2019, 59 (9), 3692–3702. 10.1021/acs.jcim.9b00470. 31361962

[ref41] Iyyer M.; Yih W.-t.; Chang M.-W. Search-based Neural Structured Learning for Sequential Question Answering. In Proceedings of the 55th Annual Meeting of the Association for Computational Linguistics (Vol. 1: Long Papers); Association for Computational Linguistics, Vancouver, Canada, 2017; pp 1821–1831, 10.18653/v1/P17-1167.

[ref42] Waskom M. L. seaborn: statistical data visualization. J. Open Source Softw. 2021, 6 (60), 3021 10.21105/joss.03021.

[ref43] Liu P.; Yuan W.; Fu J.; Jiang Z.; Hayashi H.; Neubig G. Pre-train, Prompt, and Predict: A Systematic Survey of Prompting Methods in Natural Language Processing. 2021, abs/2107.13586. ArXiv2107.13586. https://arxiv.org/abs/2107.13586 (accessed 2023-03-06).

[ref44] Jain S.; Wallace B. C. Attention is not Explanation. 2019, 1902.10186. ArXiv1902.10186. https://arxiv.org/abs/1902.10186 (accessed 2023-03-06).

