# Supplementary material for: OpticalBERT and OpticalTable-SQA: Text- and Table-Based Language Models for the Optical-Materials Domain
Source: J Chem Inf Model. 2023 Mar 20;63(7):1961–81. doi: 10.1021/acs.jcim.2c01259 (PMC10091421; doi:10.1021/acs.jcim.2c01259)
Supplement: Supplementary file 1 — ci2c01259_si_002.zip [file ci2c01259_si_002.zip › SIrev/SI_opticalbert_clean.pdf]

Supporting Information: OpticalBERT and OpticalTable-SQA: Text- and Table-based Language Models for the Optical-Materials Domain

Jiuyang Zhao[1], Shu Huang[1], and Jacqueline M. Cole[1,2,*]

[1] Cavendish Laboratory, University of Cambridge, J. J. Thomson Avenue, Cambridge, CB3 0HE, U.K.
[2] ISIS Neutron and Muon Source, Rutherford Appleton Laboratory, Harwell Science and Innovation Campus, Didcot, Oxfordshire, OX11 0QX, U.K.
[*] corresponding author: Jacqueline M. Cole (jmc61@cam.ac.uk)

Contents

1 Acronyms / Abbreviations 3

2 Implementation Details 3
- 2.1 Pre-training . . . 3
- 2.2 Fine-tuning . . . 4
  - 2.2.1 Abstract Classification . . . 4
  - 2.2.2 Question Answering . . . 4
  - 2.2.3 Chemical-Named-Entity Recognition . . . 4
  - 2.2.4 OpticalTable-SQA . . . 5

3 Evaluation details 5
  - 3.0.1 Abstract Classification . . . 5
  - 3.0.2 Question Answering . . . 8
  - 3.0.3 Chemical-Named-Entity Recognition . . . 13
  - 3.0.4 OpticalTable-SQA . . . 17

4 Evaluation files 18
- 4.1 Journal name annotation validation dataset . . . 18
- 4.2 Abstract classification test dataset . . . 18
- 4.3 Question answering arbitrary test dataset . . . 18
- 4.4 Question answering numerical test dataset . . . 18

4.5 Question answering numerical test dataset CDE 18
4.6 Chemical-named-entity recognition test dataset text 18
4.7 Chemical-named-entity recognition test dataset entities 18

1 Acronyms / Abbreviations

This section describes a list of abbreviations used in the manuscript. The order of these abbreviations in the list follows the order of those that appear in the manuscript.

- BERT: Bidirectional encoder representations from Transformers
- NLP: Natural language processing
- SQA: Sequential question answering
- CDE: ChemDataExtractor
- UV: Ultra-violet
- LSTM: Long-short-term memory (model)
- ELMo: Embeddings from Language Models
- GPT: Generative pre-trained transforme
- TableQA: Table Question Answering
- Tapas: Weakly supervised **ta**ble **pa**rsing via pre-training
- T3QA: Topic transferable table question answering
- NSP: Next sentence prediction
- RSC: Royal society of chemistry
- API: Application programming interface
- MLM: Masked-language modeling
- ALCF: Argonne leadership computing facility
- CNER: Chemical-named-entity Recognition
- LR: Logistic regression
- 1M: one million
- Tf-IDF: Term frequency-inverse document frequency
- EM: Exact match

2 Implementation Details

2.1 Pre-training

The language model utilized the BertForMaskedLM architecture implemented from the Python library transformers [1]. Some key training parameters and model configuration shared by all models are listed below:

- number of layers: 12

- hidden size: 768
- number of self-attention heads: 12
- maximum sequence length: 512
- batch size: 256
- hidden layer dropout probability: 0.1
- attention layer dropout probability: 0.1
- transformers version: 4.18.0.dev0
- optimizer: AdamW
- adam β_1: 0.9
- adam β_2: 0.999
- adam ϵ: 1e-08
- L2 weight decay: 0.01
- vocab size: 28996 (cased), 30522 (uncased)
- warmup steps: 10000

The difference in training parameters between models is the total training epoch and learning rate. OpticalBERT was trained from the initial weights of the original BERT model for 35 epochs with an initial learning rate of 2e-5. OpticalPureBERT was trained from scratch for 40 epochs with an initial learning rate of 1e-4.

2.2 Fine-tuning

2.2.1 Abstract Classification

The BertForSequenceClassification architecture implemented in transformers [1] was employed to perform the abstract classification task. We fine-tuned 8 epochs using a batch size of 16 and 32, and a learning rate of 1e-5, 2e-5 and 5e-5.

2.2.2 Question Answering

The BertForQuestionAnswering architecture implemented in transformers [1] was employed to perform the question-answering task for text. We fine-tuned 4 epochs using a batch size of 16 and 32, and a learning rate of 1e-5, 2e-5 and 5e-5.

2.2.3 Chemical-Named-Entity Recognition

The BertForTokenClassification architecture implemented in transformers [1] was employed to perform the chemical-named-entity recognition task. We fine-tuned 20 epochs using a batch size of 16 and 32, and a learning rate of 2e-5, 5e-5 and 8e-5.

2.2.4 OpticalTable-SQA

The TapasForQuestionAnswering architecture implemented in transformers [1] was employed to perform the question-answering task for tables. We fine-tuned 30 epochs using a batch size of 16 and 32, and a learning rate of 1e-6, 2e-6 and 5e-6.

3 Evaluation details

This section presents the evaluation details of all fine-tuned models mentioned in the manuscript. The performances of different models on both the development sets and the test sets are shown across 11 tables. The "SciBERT", "MatBERT" and "MatSciBERT" refer to our own versions of fine-tuned SciBERT, MatBERT and MatSciBERT models.

3.0.1 Abstract Classification

Table S1: The validation accuracy of evaluated models in the abstract classification tasks of different hyper-parameter sets. The column header "Accuracy (16, 1e-5)" indicates that this column represents the accuracy of models of a batch size of 16 and a learning rate of 1e-5. The cell value "93.80 (3)" indicates the model achieves the highest accuracy of 93.80% at epoch 3.

	Accuracy (16,1e-5)	Accuracy (16,2e-5)	Accuracy (16,5e-5)
BERT cased	93.80 (3)	94.02 (3)	92.83 (1)
SciBERT cased	94.30 (3)	94.18 (2)	93.40 (1)
OpticalBERT cased	94.27 (2)	94.11 (2)	93.55 (2)
OpticalPureBERT cased	94.84 (3)	94.69 (2)	94.08 (8)
MatBERT cased	94.26 (2)	94.33 (3)	91.91 (6)
BERT uncased	93.98 (2)	93.82 (1)	92.90 (2)
SciBERT uncased	94.48 (4)	94.50 (2)	93.26 (6)
OpticalBERT uncased	94.41 (1)	94.22 (5)	93.36 (5)
OpticalPureBERT uncased	94.82 (2)	94.62 (3)	93.96 (6)
MatBERT uncased	94.54 (2)	94.64 (2)	93.44 (2)
MatSciBERT uncased	94.58 (4)	94.33 (2)	93.50 (6)
	Accuracy (32,1e-5)	Accuracy (32,2e-5)	Accuracy (32,5e-5)
BERT cased	93.93 (2)	93.89 (2)	93.20 (4)
SciBERT cased	94.38 (3)	94.35 (2)	93.91 (1)
OpticalBERT cased	94.36 (2)	94.32 (2)	93.99 (1)
OpticalPureBERT cased	94.94 (3)	94.80 (1)	94.38 (2)
MatBERT cased	94.39 (2)	94.24 (3)	93.96 (8)
BERT uncased	93.91 (2)	94.09 (2)	93.47 (2)
SciBERT uncased	94.71 (3)	94.55 (3)	93.78 (5)
OpticalBERT uncased	94.50 (2)	94.19 (2)	94.14 (5)
OpticalPureBERT uncased	95.03 (2)	95.03 (3)	94.67 (2)
MatBERT uncased	94.53 (2)	94.49 (3)	94.20 (1)
MatSciBERT uncased	94.70 (2)	94.43 (1)	94.05 (4)

Table S2: The test precision, recall, and F1 score of evaluated models with different random weight initialization of their prediction layers in the abstract classification task.

Model	Precision	Recall	F1
BERT_base_cased/ini_1	0.806349206	0.648854962	0.719079418
BERT_base_cased/ini_2	0.806349206	0.633587786	0.709604672
BERT_base_cased/ini_3	0.812698413	0.65648855	0.726289051
BERT_base_cased/ini_4	0.812698413	0.65648855	0.726289051
BERT_base_cased/ini_5	0.815873016	0.65648855	0.727554027
BERT_base_uncased/ini_1	0.80952381	0.641221374	0.715610123
BERT_base_uncased/ini_2	0.819047619	0.648854962	0.724084988
BERT_base_uncased/ini_3	0.819047619	0.648854962	0.724084988
BERT_base_uncased/ini_4	0.815873016	0.633587786	0.71326824
BERT_base_uncased/ini_5	0.806349206	0.641221374	0.714367027
OpticalBERT_cased/ini_1	0.822222222	0.641221374	0.720528573
OpticalBERT_cased/ini_2	0.819047619	0.625954198	0.709599517
OpticalBERT_cased/ini_3	0.819047619	0.625954198	0.709599517
OpticalBERT_cased/ini_4	0.815873016	0.625954198	0.708405466
OpticalBERT_cased/ini_5	0.819047619	0.633587786	0.714478755
OpticalBERT_uncased/ini_1	0.812698413	0.618320611	0.702308174
OpticalBERT_uncased/ini_2	0.822222222	0.618320611	0.705840791
OpticalBERT_uncased/ini_3	0.80952381	0.618320611	0.701120163
OpticalBERT_uncased/ini_4	0.819047619	0.625954198	0.709599517
OpticalBERT_uncased/ini_5	0.80952381	0.610687023	0.696186332
MatBERT_cased/ini_1	0.819047619	0.633587786	0.714478755
MatBERT_cased/ini_2	0.822222222	0.633587786	0.71568399
MatBERT_cased/ini_3	0.822222222	0.625954198	0.710788333
MatBERT_cased/ini_4	0.80952381	0.618320611	0.701120163
MatBERT_cased/ini_5	0.812698413	0.610687023	0.697357668
MatBERT_uncased/ini_1	0.80952381	0.625954198	0.706001519
MatBERT_uncased/ini_2	0.815873016	0.641221374	0.718080064
MatBERT_uncased/ini_3	0.819047619	0.65648855	0.728813559
MatBERT_uncased/ini_4	0.812698413	0.641221374	0.71684779
MatBERT_uncased/ini_5	0.80952381	0.641221374	0.715610123
MatSciBERT_uncased/ini_1	0.815873016	0.618320611	0.703490926
MatSciBERT_uncased/ini_2	0.815873016	0.618320611	0.703490926
MatSciBERT_uncased/ini_3	0.80952381	0.610687023	0.696186332
MatSciBERT_uncased/ini_4	0.819047619	0.618320611	0.704668454
MatSciBERT_uncased/ini_5	0.819047619	0.610687023	0.699684735
OpticalPureBERT_cased/ini_1	0.815873016	0.618320611	0.703490926
OpticalPureBERT_cased/ini_2	0.822222222	0.633587786	0.71568399
OpticalPureBERT_cased/ini_3	0.815873016	0.633587786	0.71326824
OpticalPureBERT_cased/ini_4	0.822222222	0.633587786	0.71568399
OpticalPureBERT_cased/ini_5	0.819047619	0.633587786	0.714478755
OpticalPureBERT_uncased/ini_1	0.822222222	0.65648855	0.730067684
OpticalPureBERT_uncased/ini_2	0.815873016	0.641221374	0.718080064
OpticalPureBERT_uncased/ini_3	0.822222222	0.648854962	0.725322878
OpticalPureBERT_uncased/ini_4	0.825396825	0.664122137	0.736028634
OpticalPureBERT_uncased/ini_5	0.828571429	0.664122137	0.737288136
SciBERT_cased/ini_1	0.812698413	0.641221374	0.71684779
SciBERT_cased/ini_2	0.812698413	0.633587786	0.712052412
SciBERT_cased/ini_3	0.815873016	0.625954198	0.708405466
SciBERT_cased/ini_4	0.796825397	0.595419847	0.681554716
SciBERT_cased/ini_5	0.815873016	0.625954198	0.708405466
SciBERT_uncased/ini_1	0.819047619	0.633587786	0.714478755
SciBERT_uncased/ini_2	0.803174603	0.603053435	0.688874337
SciBERT_uncased/ini_3	0.812698413	0.603053435	0.692353777
SciBERT_uncased/ini_4	0.812698413	0.610687023	0.697357668
SciBERT_uncased/ini_5	0.80952381	0.610687023	0.696186332

3.0.2 Question Answering

Table S3: The validation accuracy of evaluated models in the question answering tasks of different hyper-parameter sets. "EM" represents exact-match score. "F1" represents the F1 score. The first column shows the model type and hyper-parameter sets.

**BERT Base**		**epoch 1**	**epoch 2**	**epoch 3**	**epoch 4**
cased, 16_1e5	EM	77.80	79.90	80.15	80.32
	F1	85.93	87.54	87.88	87.96
cased, 16_2e5	EM	79.31	80.94	81.02	80.83
	F1	86.93	88.29	88.44	88.41
cased, 16_5e5	EM	78.33	80.65	80.10	79.67
	F1	86.56	87.88	87.74	87.69
cased, 32_1e5	EM	76.49	79.19	79.61	79.87
	F1	85.16	87.06	87.37	87.59
cased, 32_2e5	EM	78.70	80.41	80.69	80.67
	F1	86.62	87.92	88.26	88.16
cased, 32_5e5	EM	79.56	81.12	80.67	80.69
	F1	87.25	88.42	88.40	88.51
uncased, 16_1e5	EM	77.17	79.12	79.86	79.82
	F1	85.46	87.10	87.62	87.55
uncased, 16_2e5	EM	79.36	80.44	80.59	80.22
	F1	87.02	87.87	88.05	87.92
uncased, 16_5e5	EM	79.71	80.07	80.41	79.80
	F1	87.18	87.63	87.64	87.90
uncased, 32_1e5	EM	75.49	77.81	78.99	78.89
	F1	84.29	86.10	86.95	86.88
uncased, 32_2e5	EM	78.24	79.29	80.33	79.75
	F1	86.28	87.30	87.92	87.62
uncased, 32_5e5	EM	79.47	79.81	80.40	79.82
	F1	87.14	87.66	88.16	87.88
**SciBERT**		**epoch 1**	**epoch 2**	**epoch 3**	**epoch 4**
cased, 16_1e5	EM	74.93	77.61	77.71	77.58
	F1	84.10	85.91	86.19	86.10
cased, 16_2e5	EM	76.73	78.46	78.20	78.24
	F1	85.36	86.71	86.73	86.60
cased, 16_5e5	EM	76.23	77.56	77.88	77.71
	F1	84.99	85.99	86.62	86.54
cased, 32_1e5	EM	73.85	76.65	77.18	77.36
	F1	83.24	85.30	85.74	85.93
cased, 32_2e5	EM	76.29	78.15	78.09	78.24
	F1	85.23	86.52	86.57	86.66
cased, 32_5e5	EM	76.79	78.06	78.29	78.19
	F1	85.63	86.21	86.75	86.69
uncased, 16_1e5	EM	76.38	78.32	78.39	78.54
	F1	85.04	86.45	86.53	86.71
uncased, 16_2e5	EM	77.78	78.82	78.90	78.92
	F1	86.17	86.97	86.86	87.15
uncased, 16_5e5	EM	76.44	77.78	78.03	77.76
	F1	84.98	86.07	86.07	86.38
uncased, 32_1e5	EM	75.53	77.42	77.91	77.91
	F1	84.53	85.95	86.13	86.14
uncased, 32_2e5	EM	77.11	78.63	78.74	78.59
	F1	85.47	86.66	86.79	86.72
uncased, 32_5e5	EM	77.43	77.93	78.51	78.25
	F1	85.80	86.11	86.58	86.71

Table S4: Table S2 continued.

**OpticalBERT**		**epoch 1**	**epoch 2**	**epoch 3**	**epoch 4**
cased, 16_1e5	EM	78.59	80.94	81.24	81.38
	F1	86.40	88.31	88.55	88.67
cased, 16_2e5	EM	79.49	81.32	81.71	81.51
	F1	87.08	88.69	88.91	88.92
cased, 16_5e5	EM	77.52	79.87	80.13	79.74
	F1	85.68	87.54	87.46	87.60
cased, 32_1e5	EM	77.47	79.42	80.33	80.70
	F1	85.80	87.36	87.81	88.12
cased, 32_2e5	EM	78.83	81.04	81.23	81.17
	F1	86.75	88.51	88.62	88.53
cased, 32_5e5	EM	78.24	81.05	81.14	80.74
	F1	86.05	88.26	88.38	88.33
uncased, 16_1e5	EM	77.29	79.09	79.54	79.79
	F1	85.93	87.23	87.54	87.85
uncased, 16_2e5	EM	78.80	80.00	79.88	79.97
	F1	86.80	88.06	87.54	87.95
uncased, 16_5e5	EM	78.86	79.16	80.18	79.54
	F1	86.45	87.42	87.66	87.55
uncased, 32_1e5	EM	75.67	77.95	78.85	79.12
	F1	84.67	86.24	87.10	87.27
uncased, 32_2e5	EM	78.05	79.35	79.94	79.83
	F1	86.33	87.44	87.72	87.75
uncased, 32_5e5	EM	78.70	79.82	80.26	79.61
	F1	86.32	87.55	87.71	87.76
**OpticalPureBERT**		**epoch 1**	**epoch 2**	**epoch 3**	**epoch 4**
cased, 16_1e5	EM	77.12	79.16	79.97	80.08
	F1	85.22	86.60	87.44	87.39
cased, 16_2e5	EM	78.12	80.63	80.07	79.93
	F1	86.20	87.71	87.74	87.55
cased, 16_5e5	EM	77.71	80.00	79.71	79.73
	F1	85.69	87.20	87.38	87.55
cased, 32_1e5	EM	76.03	78.59	78.69	79.16
	F1	84.39	86.33	86.50	86.96
cased, 32_2e5	EM	78.02	79.85	79.73	80.23
	F1	85.88	87.22	87.44	87.75
cased, 32_5e5	EM	77.77	80.00	79.86	79.57
	F1	86.21	87.25	87.29	87.35
uncased, 16_1e5	EM	77.33	78.98	79.59	79.72
	F1	85.89	86.99	87.38	87.55
uncased, 16_2e5	EM	78.25	79.63	80.02	79.51
	F1	86.34	87.56	87.66	87.47
uncased, 16_5e5	EM	76.55	78.60	78.92	78.42
	F1	85.11	86.51	86.65	86.60
uncased, 32_1e5	EM	75.94	78.13	78.70	78.93
	F1	84.90	86.38	86.71	87.02
uncased, 32_2e5	EM	77.68	79.08	79.98	79.81
	F1	86.23	87.06	87.74	87.70
uncased, 32_5e5	EM	77.11	78.67	78.91	78.86
	F1	85.81	86.84	87.20	87.34

Table S5: Table S2 continued.

**MatBERT**		**epoch 1**	**epoch 2**	**epoch 3**	**epoch 4**
cased, 16_1e5	EM	74.24	76.19	76.76	76.83
	F1	83.56	84.69	85.32	85.53
cased, 16_2e5	EM	75.54	77.18	77.54	77.33
	F1	84.47	85.38	85.97	86.01
cased, 16_5e5	EM	73.46	76.21	76.93	76.15
	F1	82.91	84.47	85.37	85.18
cased, 32_1e5	EM	72.75	75.12	76.00	76.07
	F1	82.52	84.26	84.81	84.90
cased, 32_2e5	EM	74.81	76.45	77.36	77.19
	F1	83.87	85.05	85.82	85.79
cased, 32_5e5	EM	74.91	76.51	76.98	76.32
	F1	83.84	84.92	85.26	85.16
uncased, 16_1e5	EM	75.43	77.71	77.99	78.17
	F1	84.57	86.19	86.46	86.55
uncased, 16_2e5	EM	76.61	78.28	77.98	77.73
	F1	85.20	86.41	86.46	86.37
uncased, 16_5e5	EM	75.17	77.43	77.19	77.26
	F1	84.37	85.49	85.99	86.09
uncased, 32_1e5	EM	74.91	77.27	77.61	77.67
	F1	84.44	85.73	86.11	86.21
uncased, 32_2e5	EM	76.49	78.72	78.38	78.19
	F1	85.51	86.6	86.79	86.57
uncased, 32_5e5	EM	75.69	77.36	77.52	77.29
	F1	84.59	85.48	86.07	86.046
**MatSciBERT**		**epoch 1**	**epoch 2**	**epoch 3**	**epoch 4**
uncased, 16_1e5	EM	75.17	77.08	77.87	77.79
	F1	84.17	85.68	86.16	86.19
uncased, 16_2e5	EM	76.82	78.18	78.41	78.10
	F1	85.28	86.43	86.54	86.37
uncased, 16_5e5	EM	76.49	78.09	78.21	77.71
	F1	85.04	86.00	86.41	86.29
uncased, 32_1e5	EM	73.85	76.25	76.85	77.03
	F1	83.18	85.16	85.53	85.52
uncased, 32_2e5	EM	75.82	77.47	77.84	77.54
	F1	84.60	86.13	86.16	86.0
uncased, 32_5e5	EM	76.13	77.87	78.32	77.35
	F1	84.88	86.10	86.35	85.98

Table S6: The test exact match, recall, and F1 score of evaluated models with different random weight initialization of their prediction layers in the arbitrary question-answering task, and the precision of the evaluated models in the numerical question-answering task.

Model	Exact_match	Recall	F1	Numerical_precision
BERT_base_cased/ini_1	61.46179402	84.58957828	81.83028746	0.750788644
BERT_base_cased/ini_2	61.46179402	84.58957828	81.83028746	0.750788644
BERT_base_cased/ini_3	61.46179402	84.58957828	81.83028746	0.750788644
BERT_base_cased/ini_4	61.46179402	84.58957828	81.83028746	0.750788644
BERT_base_cased/ini_5	61.46179402	84.58957828	81.83028746	0.750788644
BERT_base_uncased/ini_1	63.4551495	87.4879138	83.41191805	0.728706625
BERT_base_uncased/ini_2	63.78737542	86.86775876	83.38900706	0.76340694
BERT_base_uncased/ini_3	63.78737542	86.86775876	83.38900706	0.76340694
BERT_base_uncased/ini_4	63.78737542	86.86775876	83.38900706	0.76340694
BERT_base_uncased/ini_5	63.78737542	86.86775876	83.38900706	0.76340694
OpticalBERT_cased/ini_1	71.76079734	88.14992279	87.40180129	0.876971609
OpticalBERT_cased/ini_2	71.76079734	88.14992279	87.40180129	0.876971609
OpticalBERT_cased/ini_3	71.76079734	88.14992279	87.40180129	0.876971609
OpticalBERT_cased/ini_4	71.76079734	88.14992279	87.40180129	0.876971609
OpticalBERT_cased/ini_5	71.76079734	88.14992279	87.40180129	0.876971609
OpticalBERT_uncased/ini_1	69.10299003	87.47138781	86.46931153	0.864353312
OpticalBERT_uncased/ini_2	69.10299003	87.47138781	86.46931153	0.864353312
OpticalBERT_uncased/ini_3	69.10299003	87.47138781	86.46931153	0.864353312
OpticalBERT_uncased/ini_4	69.10299003	87.47138781	86.46931153	0.864353312
OpticalBERT_uncased/ini_5	69.10299003	87.47138781	86.46931153	0.864353312
MatBERT_cased/ini_1	66.11295681	89.32739166	86.88083001	0.842271293
MatBERT_cased/ini_2	66.11295681	89.32739166	86.88083001	0.842271293
MatBERT_cased/ini_3	66.11295681	89.32739166	86.88083001	0.842271293
MatBERT_cased/ini_4	66.11295681	89.32739166	86.88083001	0.842271293
MatBERT_cased/ini_5	66.11295681	89.32739166	86.88083001	0.842271293
MatBERT_uncased/ini_1	68.10631229	90.26632537	86.97859542	0.861198738
MatBERT_uncased/ini_2	68.10631229	90.26632537	86.97859542	0.861198738
MatBERT_uncased/ini_3	68.10631229	90.26632537	86.97859542	0.861198738
MatBERT_uncased/ini_4	68.10631229	90.26632537	86.97859542	0.861198738
MatBERT_uncased/ini_5	68.10631229	90.26632537	86.97859542	0.861198738
MatSciBERT_uncased/ini_1	69.10299003	90.8513522	88.31754662	0.851735016
MatSciBERT_uncased/ini_2	69.10299003	90.8513522	88.31754662	0.851735016
MatSciBERT_uncased/ini_3	69.10299003	90.8513522	88.31754662	0.851735016
MatSciBERT_uncased/ini_4	69.10299003	90.8513522	88.31754662	0.851735016
MatSciBERT_uncased/ini_5	69.10299003	90.8513522	88.31754662	0.851735016
OpticalPureBERT_cased/ini_1	70.09966777	89.64617033	87.02173421	0.867507886
OpticalPureBERT_cased/ini_2	70.09966777	89.64617033	87.02173421	0.867507886
OpticalPureBERT_cased/ini_3	70.09966777	89.64617033	87.02173421	0.867507886
OpticalPureBERT_cased/ini_4	70.09966777	89.64617033	87.02173421	0.867507886
OpticalPureBERT_cased/ini_5	70.09966777	89.64617033	87.02173421	0.867507886
OpticalPureBERT_uncased/ini_1	73.75415282	89.84199567	88.67050293	0.873817035
OpticalPureBERT_uncased/ini_2	73.75415282	89.84199567	88.67050293	0.873817035
OpticalPureBERT_uncased/ini_3	73.75415282	89.84199567	88.67050293	0.873817035
OpticalPureBERT_uncased/ini_4	73.75415282	89.84199567	88.67050293	0.873817035
OpticalPureBERT_uncased/ini_5	73.75415282	89.84199567	88.67050293	0.873817035
SciBERT_cased/ini_1	66.44518272	88.21029724	85.22523404	0.760252366
SciBERT_cased/ini_2	66.44518272	88.21029724	85.22523404	0.760252366
SciBERT_cased/ini_3	66.44518272	88.21029724	85.22523404	0.760252366
SciBERT_cased/ini_4	66.44518272	88.21029724	85.22523404	0.760252366
SciBERT_cased/ini_5	66.44518272	88.21029724	85.22523404	0.760252366
SciBERT_uncased/ini_1	67.44186047	88.82812994	86.44717221	0.842271293
SciBERT_uncased/ini_2	67.44186047	88.82812994	86.44717221	0.842271293
SciBERT_uncased/ini_3	67.44186047	88.82812994	86.44717221	0.842271293
SciBERT_uncased/ini_4	67.44186047	88.82812994	86.44717221	0.842271293
SciBERT_uncased/ini_5	67.44186047	88.82812994	86.44717221	0.842271293

3.0.3 Chemical-Named-Entity Recognition

Table S7: The validation precision, recall and F1 score of evaluated models in the chemical named entity recognition tasks of different hyper-parameter sets. The first column shows the model type and hyper-parameter sets.

**BERT Base**	**Precision**	**Recall**	**F1**	**Best epoch**
cased 16_2e5	0.8694	0.9204	0.8941	20
cased 16_5e5	0.8851	0.9220	0.9032	20
cased 16_8e5	0.8869	0.9238	0.9050	20
cased 32_2e5	0.8523	0.9084	0.8795	20
cased 32_5e5	0.8794	0.9161	0.8974	19
cased 32_8e5	0.8875	0.9214	0.9042	20
uncased 16_2e5	0.8900	0.9248	0.9071	18
uncased 16_5e5	0.8957	0.9296	0.9124	19
uncased 16_8e5	0.9004	0.9292	0.9146	19
uncased 32_2e5	0.8890	0.9284	0.9083	20
uncased 32_5e5	0.8951	0.9322	0.9133	19
uncased 32_8e5	0.8974	0.9280	0.9124	19
**SciBERT**	**Precision**	**Recall**	**F1**	**Best epoch**
cased 16_2e5	0.9028	0.9372	0.9197	15
cased 16_5e5	0.9140	0.9308	0.9223	16
cased 16_8e5	0.9072	0.9333	0.9201	19
cased 32_2e5	0.8990	0.9337	0.9160	18
cased 32_5e5	0.9069	0.9383	0.9223	18
cased 32_8e5	0.9074	0.9375	0.9222	19
uncased 16_2e5	0.9083	0.9356	0.9218	17
uncased 16_5e5	0.9123	0.9389	0.9254	19
uncased 16_8e5	0.9095	0.9392	0.9241	17
uncased 32_2e5	0.9048	0.9398	0.9220	17
uncased 32_5e5	0.9102	0.9422	0.9259	20
uncased 32_8e5	0.9104	0.9416	0.9257	20

Table S8: Table S4 continued.

**OpticalBERT**	**Precision**	**Recall**	**F1**	**Best epoch**
cased 16_2e5	0.9091	0.9399	0.9243	19
cased 16_5e5	0.9141	0.9384	0.9261	18
cased 16_8e5	0.9099	0.9373	0.9234	19
cased 32_2e5	0.9079	0.9388	0.9231	19
cased 32_5e5	0.9118	0.9399	0.9257	20
cased 32_8e5	0.9132	0.9384	0.9256	20
uncased 16_2e5	0.9095	0.9373	0.9232	20
uncased 16_5e5	0.9118	0.9401	0.9257	20
uncased 16_8e5	0.9120	0.9367	0.9242	16
uncased 32_2e5	0.9049	0.9396	0.9219	20
uncased 32_5e5	0.9127	0.9433	0.9278	20
uncased 32_8e5	0.9126	0.9381	0.9252	19
**OpticalPureBERT**	**Precision**	**Recall**	**F1**	**Best epoch**
cased 16_2e5	0.9131	0.9445	0.9286	16
cased 16_5e5	0.9104	0.9419	0.9259	15
cased 16_8e5	0.9207	0.9410	0.9308	18
cased 32_2e5	0.9125	0.9407	0.9264	18
cased 32_5e5	0.9115	0.9406	0.9258	15
cased 32_8e5	0.9152	0.9396	0.9272	19
uncased 16_2e5	0.9094	0.9463	0.9275	17
uncased 16_5e5	0.9091	0.9416	0.9250	20
uncased 16_8e5	0.9101	0.9384	0.9240	20
uncased 32_2e5	0.9070	0.9402	0.9233	20
uncased 32_5e5	0.9082	0.9431	0.9253	17
uncased 32_8e5	0.9082	0.9410	0.9243	20

Table S9: Table S4 continued.

**MatBERT**	**Precision**	**Recall**	**F1**	**Best epoch**
cased 16_2e5	0.9055	0.9357	0.9203	18
cased 16_5e5	0.9100	0.9368	0.9232	20
cased 16_8e5	0.9122	0.9379	0.9249	20
cased 32_2e5	0.9029	0.9347	0.9185	20
cased 32_5e5	0.9063	0.9361	0.9210	14
cased 32_8e5	0.9082	0.9369	0.9223	19
uncased 16_2e5	0.9060	0.9452	0.9252	14
uncased 16_5e5	0.9110	0.9397	0.9251	18
uncased 16_8e5	0.9107	0.9386	0.9244	19
uncased 32_2e5	0.9038	0.9434	0.9230	20
uncased 32_5e5	0.9072	0.9433	0.9249	14
uncased 32_8e5	0.9066	0.9383	0.9222	20
**MatSciBERT**	**Precision**	**Recall**	**F1**	**Best epoch**
uncased 16_2e5	0.9060	0.9411	0.9233	19
uncased 16_5e5	0.9114	0.9378	0.9244	20
uncased 16_8e5	0.9066	0.9387	0.9223	20
uncased 32_2e5	0.9043	0.9377	0.9207	10
uncased 32_5e5	0.9101	0.9426	0.9260	15
uncased 32_8e5	0.9081	0.9392	0.9234	19

Table S10: The test precision, recall, and F1 score of evaluated models with different random weight initialization of their prediction layers in the chemical named entity recognition task.

Model	Precision	Recall	F1
BERT_base_cased/ini_1	0.811735941	0.832080201	0.821782178
BERT_base_cased/ini_2	0.811735941	0.832080201	0.821782178
BERT_base_cased/ini_3	0.811735941	0.832080201	0.821782178
BERT_base_cased/ini_4	0.811735941	0.832080201	0.821782178
BERT_base_cased/ini_5	0.811735941	0.832080201	0.821782178
BERT_base_uncased/ini_1	0.781407035	0.779448622	0.7804266
BERT_base_uncased/ini_2	0.781407035	0.779448622	0.7804266
BERT_base_uncased/ini_3	0.781407035	0.779448622	0.7804266
BERT_base_uncased/ini_4	0.781407035	0.779448622	0.7804266
BERT_base_uncased/ini_5	0.781407035	0.779448622	0.7804266
OpticalBERT_cased/ini_1	0.787128713	0.796992481	0.792029888
OpticalBERT_cased/ini_2	0.787128713	0.796992481	0.792029888
OpticalBERT_cased/ini_3	0.787128713	0.796992481	0.792029888
OpticalBERT_cased/ini_4	0.787128713	0.796992481	0.792029888
OpticalBERT_cased/ini_5	0.787128713	0.796992481	0.792029888
OpticalBERT_uncased/ini_1	0.809045226	0.807017544	0.808030113
OpticalBERT_uncased/ini_2	0.809045226	0.807017544	0.808030113
OpticalBERT_uncased/ini_3	0.809045226	0.807017544	0.808030113
OpticalBERT_uncased/ini_4	0.809045226	0.807017544	0.808030113
OpticalBERT_uncased/ini_5	0.809045226	0.807017544	0.808030113
MatBERT_cased/ini_1	0.767088608	0.759398496	0.763224181
MatBERT_cased/ini_2	0.767088608	0.759398496	0.763224181
MatBERT_cased/ini_3	0.767088608	0.759398496	0.763224181
MatBERT_cased/ini_4	0.767088608	0.759398496	0.763224181
MatBERT_cased/ini_5	0.767088608	0.759398496	0.763224181
MatBERT_uncased/ini_1	0.777239709	0.804511278	0.790640394
MatBERT_uncased/ini_2	0.777239709	0.804511278	0.790640394
MatBERT_uncased/ini_3	0.777239709	0.804511278	0.790640394
MatBERT_uncased/ini_4	0.777239709	0.804511278	0.790640394
MatBERT_uncased/ini_5	0.777239709	0.804511278	0.790640394
MatSciBERT_uncased/ini_1	0.78817734	0.802005013	0.795031056
MatSciBERT_uncased/ini_2	0.78817734	0.802005013	0.795031056
MatSciBERT_uncased/ini_3	0.78817734	0.802005013	0.795031056
MatSciBERT_uncased/ini_4	0.78817734	0.802005013	0.795031056
MatSciBERT_uncased/ini_5	0.78817734	0.802005013	0.795031056
OpticalPureBERT_cased/ini_1	0.820638821	0.837092732	0.828784119
OpticalPureBERT_cased/ini_2	0.820638821	0.837092732	0.828784119
OpticalPureBERT_cased/ini_3	0.820638821	0.837092732	0.828784119
OpticalPureBERT_cased/ini_4	0.820638821	0.837092732	0.828784119
OpticalPureBERT_cased/ini_5	0.820638821	0.837092732	0.828784119
OpticalPureBERT_uncased/ini_1	0.793269231	0.827067669	0.809815951
OpticalPureBERT_uncased/ini_2	0.793269231	0.827067669	0.809815951
OpticalPureBERT_uncased/ini_3	0.793269231	0.827067669	0.809815951
OpticalPureBERT_uncased/ini_4	0.793269231	0.827067669	0.809815951
OpticalPureBERT_uncased/ini_5	0.793269231	0.827067669	0.809815951
SciBERT_cased/ini_1	0.79009434	0.839598997	0.814094775
SciBERT_cased/ini_2	0.79009434	0.839598997	0.814094775
SciBERT_cased/ini_3	0.79009434	0.839598997	0.814094775
SciBERT_cased/ini_4	0.79009434	0.839598997	0.814094775
SciBERT_cased/ini_5	0.79009434	0.839598997	0.814094775
SciBERT_uncased/ini_1	0.776699029	0.802005013	0.789149199
SciBERT_uncased/ini_2	0.776699029	0.802005013	0.789149199
SciBERT_uncased/ini_3	0.776699029	0.802005013	0.789149199
SciBERT_uncased/ini_4	0.776699029	0.802005013	0.789149199
SciBERT_uncased/ini_5	0.776699029	0.802005013	0.789149199

3.0.4 OpticalTable-SQA

Table S11: The validation accuracy of different splits in the table question-answering tasks of different hyper-parameter sets. The first column shows the split and hyper-parameter sets. "SQA 1" represents the accuracy of the 1st question within the SQA validation dataset. "What" represents the accuracy of the what-question within the OpticalTableQA validation set.

**Split 1**	**SQA 1**	**SQA 2**	**SQA 3**	**SQA Overall**	**What**	**Which**	**Overall**
16_1e6	0.7892	0.6669	0.5973	0.6763	0.8669	0.8975	0.8810
16_2e6	0.7854	0.6650	0.5930	0.6723	0.8914	0.9098	0.9000
16_5e6	0.7785	0.6552	0.5929	0.6643	0.9194	0.9077	0.9140
32_1e6	0.7873	0.6611	0.5871	0.6713	0.8493	0.8893	0.8677
32_2e6	0.7873	0.6650	0.5885	0.6729	0.8774	0.9057	0.8904
32_5e6	0.7834	0.6621	0.5871	0.6673	0.89842	0.9159	0.9065
**Split 1**	**SQA 1**	**SQA 2**	**SQA 3**	**SQA Overall**	**What**	**Which**	**Overall**
16_1e6	0.7854	0.6641	0.5944	0.6730	0.7413	0.7756	0.7578
16_2e6	0.7824	0.6582	0.5944	0.6683	0.7861	0.7792	0.7828
16_5e6	0.7785	0.6621	0.5900	0.6673	0.8010	0.8654	0.8319
32_1e6	0.7844	0.6572	0.5886	0.6687	0.7148	0.7504	0.7319
32_2e6	0.7863	0.6563	0.5900	0.6680	0.7778	0.7774	0.7776
32_5e6	0.7815	0.6611	0.5974	0.6693	0.7828	0.8330	0.8069
**Split 3**	**SQA 1**	**SQA 2**	**SQA 3**	**SQA Overall**	**What**	**Which**	**Overall**
16_1e6	0.7883	0.6611	0.5930	0.6730	0.8342	0.8824	0.8567
16_2e6	0.7893	0.6670	0.5944	0.6750	0.8806	0.8763	0.8786
16_5e6	0.7776	0.6611	0.6018	0.6703	0.8859	0.8945	0.88899
32_1e6	0.7883	0.6641	0.5886	0.6726	0.7932	0.8722	0.8302
32_2e6	0.7883	0.6660	0.5959	0.6749	0.8681	0.8742	0.8710
32_5e6	0.7854	0.6660	0.5974	0.6733	0.8787	0.8904	0.8842
**Split 4**	**SQA 1**	**SQA 2**	**SQA 3**	**SQA Overall**	**What**	**Which**	**Overall**
16_1e6	0.7893	0.6631	0.5944	0.6740	0.7165	0.8004	0.7556
16_2e6	0.7873	0.6563	0.5886	0.6703	0.7617	0.8363	0.7965
16_5e6	0.7785	0.6592	0.5974	0.6697	0.7722	0.8782	0.8216
32_1e6	0.7863	0.6572	0.5827	0.6689	0.6904	0.7884	0.7360
32_2e6	0.7844	0.6582	0.5886	0.6679	0.7391	0.7944	0.7648
32_5e6	0.7775	0.6533	0.5841	0.6623	0.7756	0.8782	0.8234
**Split 5**	**SQA 1**	**SQA 2**	**SQA 3**	**SQA Overall**	**What**	**Which**	**Overall**
16_1e6	0.7863	0.6650	0.5900	0.6723	0.7784	0.7897	0.7837
16_2e6	0.7834	0.6641	0.5842	0.6709	0.8167	0.8531	0.8337
16_5e6	0.7775	0.6582	0.5754	0.6614	0.8272	0.8670	0.8458
32_1e6	0.7824	0.6562	0.5797	0.6646	0.7609	0.7757	0.7678
32_2e6	0.7814	0.6621	0.5871	0.6693	0.7940	0.7936	0.7938
32_5e6	0.7805	0.6630	0.5885	0.6669	0.8219	0.8551	0.8375

4 Evaluation files

4.1 Journal name annotation validation dataset

See separate file.

4.2 Abstract classification test dataset

See separate file.

4.3 Question answering arbitrary test dataset

See separate file.

4.4 Question answering numerical test dataset

See separate file.

4.5 Question answering numerical test dataset CDE

See separate file.

4.6 Chemical-named-entity recognition test dataset text

See separate file.

4.7 Chemical-named-entity recognition test dataset entities

See separate file.

References

[1] Wolf, T. *et al.* Huggingface's transformers: State-of-the-art natural language processing. *CoRR* **abs/1910.03771** (2019). URL `http://arxiv.org/abs/1910.03771`. `1910.03771`.
